# Three-dimensional modelling of lymphangiogenesis in-vitro using bioorthogonal click-crosslinked gelatin hydrogels

**DOI:** 10.1016/j.mtbio.2025.102367

**Published:** 2025-10-01

**Authors:** Dana E. Al-Ansari, Yangshuo Hu, Nicola Contessi Negrini, Daisy Jones, Graeme M. Birdsey, Adam D. Celiz

**Affiliations:** a National Heart and Lung Institute, Imperial College London, London, W12 0NN, UK; b Department of Bioengineering, Imperial College London, London, W12 0BZ, UK; c The Francis Crick Institute, London, NW1 1AT, UK

**Keywords:** Lymphangiogenesis, Lymphatic endothelial cells, Click chemistry hydrogels, Tissue engineering, 3D *in vitro* models, Growth factor delivery

## Abstract

Lymphangiogenesis, the formation of new lymphatic vessels from pre-existing vessels, is crucial for maintaining tissue homeostasis and immune function. Despite recent advances in understanding the molecular mechanisms regulating lymphangiogenesis, most *in vitro* studies rely on traditional two-dimensional (2D) cell cultures, with limited replication of the complex microenvironment that governs lymphangiogenesis *in vivo*. Here, we present a three-dimensional (3D) lymphangiogenesis model using gelatin hydrogels modified with click-chemistry motifs (tetrazine and norbornene, GelTN), providing a biomimetic and mechanically tunable extracellular matrix (ECM) for lymphatic endothelial cells. By encapsulating human dermal lymphatic endothelial cells (HDLEC) spheroids in GelTN, we established a robust and reliable *in vitro* sprouting assay (<48 h duration) to investigate the effects of GelTN stiffness on lymphangiogenesis. HDLEC encapsulated in low GelTN concentrations exhibited enhanced sprouting in response to vascular endothelial growth factor (VEGF)-C stimulation, compared to HDLEC encapsulated in higher GelTN concentrations. We also provide evidence for the involvement of β3 integrin in lymphangiogenesis. The reduced sprout length upon β3 integrin inhibition further decreased with combined inhibition of α5β1, suggesting a synergistic interaction of the integrin subunits in controlling HDLEC-ECM mechanotransduction. GelTN hydrogels were also evaluated for their translational potential, demonstrating sustained release of VEGF-C *in vitro* and supporting cellular infiltration and neo-vessel formation following subcutaneous injection in an *in vivo* mouse model. Overall, these findings highlight the versatility of GelTN hydrogels as a platform for studying lymphangiogenesis and their potential use for therapeutic applications that require controlled growth factor delivery in tissue engineering and regenerative medicine.

## Graphical abstract

**Figure:**
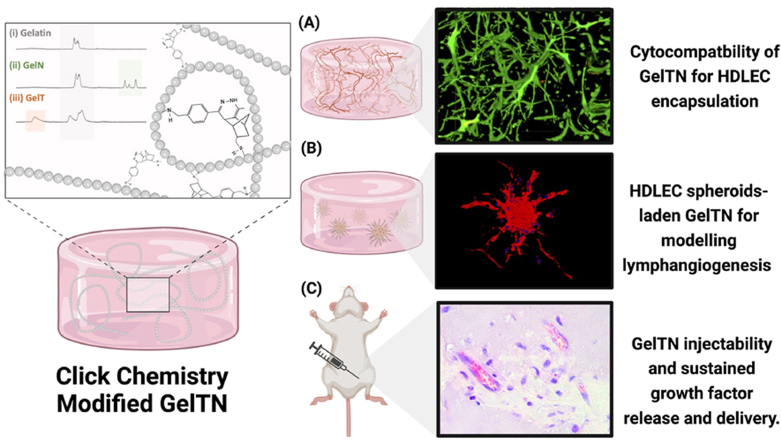

## 1 Introduction

Lymphangiogenesis is the process of lymphatic vessel development from pre-existing vessels. Lymphatic vessels form interconnected networks that are extended to most tissues, which serve their function in maintaining interstitial fluid homeostasis, tissue hydrostatic pressure and immune surveillance [1,2]. During development, lymphangiogenesis is mainly driven by the vascular endothelial growth factor C (VEGF-C)/VEGF receptor 3 (VEGFR3) signalling pathway, upon the differentiation of lymphatic endothelial cells (LEC) from the embryonic anterior cardinal vein [1,3]. In adults, lymphangiogenesis is quiescent, with the exception of some pathological conditions, such as inflammation and tumour metastasis [4]. Recent studies have uncovered the importance of VEGF-C in stimulating lymphangiogenesis for resolving inflammation and accelerating wound healing in chronic diseases, such as lymphedema [5,6], and cardiovascular diseases (CVDs), such as myocardial infarction (MI) [[7], [8], [9]]. Despite previous efforts to understand the cellular and molecular mechanisms that underlie the formation of lymphatic vessels and drive lymphangiogenesis, most *in vitro* studies predominantly focus on the role of growth factors in stimulating lymphangiogenesis by employing two-dimensional (2D) monolayer cell cultures, which limit the potential for understanding these complex processes [[10], [11], [12], [13]]. These approaches generally rely on unconstrained cell migration, forced apical polarity and restricted adhesion to x – y planes, and therefore, they do not accurately mimic the complex cellular behaviour in their physiological niches [14,15]. Given that the extracellular matrix (ECM) provides key mechanical cues such as stiffness and viscoelasticity, which are critical regulators of angiogenesis and lymphangiogenesis, recapitulating these properties is essential for understanding LEC behaviour [[16], [17], [18]].

Hydrogels offer a promising platform for 3D *in vitro* modelling by enhancing cell–ECM interactions, inducing physical constraints on the cells, and influencing the spatial organization of cell surface receptors without applying forced polarity [14,15,19]. Gelatin hydrogels provide an attractive option for *in vitro* modelling due to their high versatility, availability, and biocompatibility. As gelatin is obtained via the partial hydrolysis of collagen, it maintains collagen sequences that are responsible for cellular interactions, such as cell-adhesion (arginine-glycine-aspartic acid; RGD) and matrix metalloproteinases (MMPs) motifs, with the advantage of being less immunogenic compared to collagen [[20], [21], [22]]. Moreover, it has been shown that gelatin has solely pro-angiogenic activity, unlike some collagen variants that contain potent anti-angiogenic amino acid sequences that release endostatin and neostatin peptides, which have inhibitory effects on endothelial cell migration and proliferation [[23], [24], [25]].

To address the associated mechanical weakness and thermal instability of gelatin, several crosslinking methods have been used to achieve enhanced physical and mechanical hydrogel properties [26,27]. Among these, bioorthogonal click-chemistry stands out by providing enhanced mechanical stability without affecting cytocompatibility, allowing precise control over network formation, and crosslinking density, without the need for external stimuli [28]. The functionalisation of gelatin with bioorthogonal click chemistry motifs, tetrazine and norbornene, has been explored previously [29]. The use of click chemistry to modify gelatin polymers offers several advantages, including providing tunable mesh sizes, tailored physico-mechanical properties, and enhanced injectability [30,31]. Moreover, bioorthogonal crosslinking ensures that the crosslinking process does not interfere with biological processes [30]. We previously optimised different degrees of modifications and the stoichiometry of gelatin-tetrazine (GelT) and gelatin-norbornene (GelN), and identified optimal formulations for tissue engineering applications [32].

Here, we leverage the tunable nature of click chemistry-modified gelatin hydrogels (GelTN) to construct a 3D *in vitro* model of lymphangiogenesis using human dermal lymphatic endothelial cell (HDLEC) spheroids. The structure of HDLEC spheroids enables the investigation of intricate cell-cell interactions and the influence of microenvironmental factors on lymphangiogenesis [33]. Using the GelTN model, we observed distinct and profound differences in the sprouting of HDLEC at varying mechanical properties. Notably, this model demonstrated, for the first time, the involvement of β3 integrin in driving sprouting behaviour of HDLEC. Additionally, we investigated the potential of using GelTN hydrogels for encapsulating and sustaining the release of growth factors for lymphangiogenesis *in vivo*, thus providing insights into the applicability of this delivery system for tissue engineering and regenerative medicine applications.

## 2 Experimental methods

### 2.1 Synthesis of gelatin derivatives

Gelatin derivatives were prepared by functionalising the gelatin backbone with methyl-tetrazine-amine (tetrazine; BroadPharm®) or 5-norbornene-2-methylamine (norbornene; TCI Chemicals) as previously described [29,32]. Briefly, gelatin was dissolved to 1 % (w/v) in 2-(N-Morpholino)-ethane-sulfonic acid (MES) buffer (pH 6, 0.1 M, Merk) at 37 °C. For the tetrazine derivative, 0.5 mmol of tetrazine per gram of gelatin was prepared by adding a molar ratio of 1:4:2 of tetrazine, N-(3-Dimethylaminopropyl)-N′- ethylcarbodiimide hydrochloride (EDC, Apollo Scientific) and N-Hydroxysuccinimide (NHS, Merk) to gelatin. For the norbornene derivative, 1 mmol of norbornene per gram of gelatin was prepared by adding a molar ratio of 1:2:1 of norbornene, EDC and NHS to gelatin. Both reactions were incubated with stirring for 4 h at 37 °C, followed by the addition of 1:1 volume of Milli-Q water under stirring for 30 min to stop the reaction. Gelatin derivative solutions were dialyzed against Milli-Q water using 3.5 KDa dialysis tubing (Spectrum™ Labs, Spectra/Por, California, US) for 4 days. Functionalised gelatin derivatives were then filtered using 0.22 μm pore filters (Merck, Millipore), lyophilized, and stored at 4^o^C until further use.

### 2.2 Proton nuclear magnetic resonance spectroscopy (^1^H NMR) analysis for GelT and GelN

To determine the degree of modification (DOM) of gelatin functionalisation with either tetrazine or norbornene, 3 % w/v gelatin (GelT or GelN) was dissolved in deuterium oxide with 0.05 % of TMSP as internal standard for NMR analysis. Using Bruker Avance 500 mHz spectrometer at 37^o^C, 256 scans and 5s delay, NMR spectra were obtained and processed using MestReNova (V.14, Mestrelab). By integrating the characteristic tetrazine and norbornene peaks in the ^1^H NMR spectra, the DOM was then calculated using the following equation [1]:

[1] $$DOM=\frac{\int molecule\;}{\int TMSP}\times \frac{9H}{2H}\times \frac{n\left(TMSP\right)\left[mmol\right]}{m\left(gelatin\right)\left(g\right)}$$

Where, ∫TMSP is the reference signal (0 ppm/integrating for 9 protons), and ∫molecule is the signal detected for norbornene (6.3–5.9 ppm/integrating for 2 protons) in GelN and tetrazine (8.5–8 ppm/integrating for 2 protons) in GelT. The n(TMSP) corresponds to the moles of TMSP, and m(gelatin) corresponds to the mass of gelatin in the tested sample, respectively. The DOM was then used to calculate the percentage DOM as percentage ratio of norbornene or tetrazine to the total amount of carboxyl groups in gelatin.

### 2.3 Rheological properties

The crosslinking kinetics of gelatin hydrogel precursors were evaluated upon mixing using oscillatory time-sweep rheological test using Torque Rheometer Kinexsus Ultra+ (NETZSCH-Gerätebau GmbH). GelT and GelN were mixed in 1:2 vol ratio, to final concentrations of 6, 8, 10 and 12 % (w/v), and directly loaded onto the rheometer plate. The test was run for 2 h at 37 °C, 1 % oscillatory force and 1 Hz frequency using parallel plate geometry (⌀ = 25 mm). Humidified conditions were maintained by using a closed chamber system, to prevent evaporation. The storage (G′) and loss (G″) moduli were recorded every 5 s throughout the rheological test run. The maximum storage modulus G'max and maximum loss modulus G’’max, time to 50 % G′ plateau (t_50 %_), and gelation time (calculated as the point where tan (d) = 1), were obtained from the G′ and G″ curves.

### 2.4 Swelling and weight variation

Gelatin hydrogel precursor solutions were prepared by mixing a 1:2 vol ratio of GelT and GelN, respectively, at final concentrations of 6, 8, 10, and 12 % (w/v). The gelatin hydrogel precursor solutions were then cast into disc-shaped PDMS moulds (⌀ = 6 mm) and allowed to crosslink for 2 h at 37 °C. The crosslinked GelTN hydrogels were lyophilized, and the initial dry weight (w_0_) was measured. GelTN hydrogels were then transferred into 24 well-plates and immersed in 1.5 mL phosphate buffered saline (PBS; PH 7.0, Sigma Aldrich), to be incubated at 37 °C, with PBS changed every second day. At each time point for up to 21 days, the supernatant was removed, and the hydrogels were weighed to obtain their swollen weight (w_t_). The weight variation Δw [%] was calculated as [2]:

[2] $$\Delta w\;\left[\%\right]=\frac{w_{t}-w_{0}}{w_{0}}\times 100$$

### 2.5 Compression testing

Unconfined compression testing of freshly prepared and 96 h swollen GelTN hydrogels (6 and 12 %; after reaching the swelling equilibrium) was performed using a 10 N load cell (Instron 5900R). The hydrogels were compressed by a single hysteresis cycle of loading and unloading at 5 % min^−1^ with a preload value of 0.0005 N and an endpoint of 30 % strain. The maximum stress *s*_max_ achieved and the Young's modulus (*E*), calculated as the slope of the linear interpolation in the 0–5 % strain, were obtained using the stress – strain (σ – ε) curves.

### 2.6 Degradation assays

To evaluate the biodegradability of GelTN, hydrogels were prepared in disc-shaped PDMS moulds and then freeze-dried to measure their initial weight (w_i_). The dry GelTN samples were then immersed in 1 U mL^−1^ collagenase type I (Sigma Aldrich) solution, prepared in PBS (PH 7, 1 mM Ca^2+^; Sigma Aldrich) and incubated at 37 °C. GelTN hydrogels immersed in PBS only was used as a negative control. The residual weight of the gelatin hydrogels was calculated as the final weight (w_f_) measured after each time-point (0, 6, 24, 30, 48, 54, 72 and final endpoint) using the below equation [3]:

[3] $$Residual\;weight\;\left[\%\right]=\frac{w_{f}}{w_{i}}\times 100$$

### 2.7 Diffusion rate

GelTN hydrogels were immersed in 2 mg/mL fluorescein isothiocyanate-Dextran (FITC-Dex, 20 KDa; Sigma-Aldrich) and incubated at different timepoints at 37 °C. At each time-point, GelTN hydrogels were transferred into a new well and digested in 1 mL collagenase type I (4 mg/mL). After 45 min, by which time point the hydrogels were fully degraded, the absorbance was measured using a CLARIOstar Plus Microplate Reader (BMG LabTech) at an excitation and emission *λ* of 483 and 530 nm, respectively. GelTN_Lo (6 % w/v) and GelTN_Hi (12 % w/v) in collagenase type I solution were set as the controls/blanks.

### 2.8 Mesh size

GelTN hydrogel mesh size was estimated as previously described [29,34,35], using the following equation [4] based on an idealized elastic network solution:

[4] $$\xi =\sqrt[{3}]{\frac{6M_{c}}{{\pi c\;N}_{AV}}}$$

Where M_c_ is the average molecular weight of a polymer strand between crosslinks, N_AV_ is Avogadro's number and c is the polymer concentration. In a low frequency regime, the storage shear modulus, G, is given by the following equation [5], based on the theory of rubber elasticity:

[5] $$G=\frac{c}{M_{c}}RT$$

The average mesh size, ξ, can therefore be estimated with known polymer concentration (c), temperature (T) and measured storage modulus (G).

### 2.9 Injectability test

The injectability of GelTN_Lo was assessed quantitively by measuring the injection force using an Instron5966 mechanical testing system. The experimental set up and optimisation was adapted from Chen et al. [36]. Briefly, 1 mL syringe fitted with a 26G needle (13 mm × 0.45 mm; Fisher Scientific) was loaded with 1 mL of GelTN_Lo and clamped into the Instron5966 mechanical testing system. Syringes were incubated at room temperature for 20 min to achieve gelation time (G’ > G″). The force of injection was measured at a flow rate of 1 mL/11 s, which mimics subcutaneous injection speed [37]. To ensure consistency, the flow rate units were converted to displacement per time (5.88 mm/s). Force measurements were taken every 5 min post gelation-time for up to 45 min. As a control, a 1 mL syringe with a 26G needle loaded with 1 mL of Milli-Q water was used.

### 2.10 Cell culture

Primary human dermal lymphatic endothelial cells (HDLEC) isolated from single donor juvenile foreskin, were purchased from PromoCell® (Heidelberg, Germany). HDLEC were cultured at 37 °C, 5 % CO_2_ and maintained in Endothelial Cell Growth Medium MV2 (EGM MV2; PromoCell®) supplemented with 5 % (v/v) fetal calf serum and EGM MV2 supplement mix (PromoCell®, Heidelberg, Germany). HDLEC were cultured up to passage 7.

### 2.11 Embedding of HDLEC in GelTN hydrogels

Cell-laden gelatin hydrogels were prepared by mixing HDLEC (2 × 10^6^ cells/mL) in gelatin derivatives, previously sterile-filtered (⌀ = 0.22 μm) and dissolved in complete EGM MV2. Gelatin hydrogel-HDLEC mixtures were prepared at different GelTN concentrations (6–12 %) and incubated according to their gelation time to initiate crosslinking. HDLEC-laden hydrogels were then cast onto sterile disc-shaped polydimethylsiloxane (PDMS) moulds (80 μL each) and incubated for an additional 2 h (37 °C, 5 % CO_2_) to assure complete cross-linking. Disc-shaped HDLEC-laden hydrogels were then moved onto a 24-well plate containing complete EGM MV2 medium.

### 2.12 Viability live/dead assay

Cells were embedded in disc-shaped GelTN hydrogels, and incubated for 1, 5 and 15 days with a media change every third day. On the day of imaging, cells were washed with Hanks’ balanced salt solution (HBSS), followed by the addition of 1 μM of Calcein-AM (Sigma Aldrich) and 5 μM propidium iodide (PI; Sigma Aldrich) and then incubated for 40 min at 37 °C, 5 % CO_2_. HDLEC-laden GelTN hydrogels were imaged using a Leica SP8 confocal microscope at a magnification of 10X using 2 fluorescent channels to image Calcein-AM (green; *λ*_exc_ = 493 nm, *λ*_ems_ = 514 nm) and PI (red; *λ*_exc_ = 560 nm, *λ*_ems_ = 619 nm). Live cells were quantified using NIH ImageJ [38,39], and presented as percentage of live cells/total number of cells x 100. The lymphatic network was quantified using ImageJ plugin Angiogenesis Analyser Tool, obtaining total network length, number of nodes, branches, segments and isolated segments [38,40].

### 2.13 Metabolic activity

The metabolic activity of HDLEC embedded in GelTN hydrogels was assessed using AlamarBlue™ Cell Viability Reagent (ThermoFisher Scientific). Samples were incubated for 1, 5, 10 and 14 days, and the media was changed every 3 days. At each timepoint, the media was replaced with 700 μL of 10 % (v/v) AlamarBlue in EGM MV2 and incubated for 4 h at 37 °C, 5 % CO_2_. After incubation, the fluorescence was recorded using a CLARIOstar Plus Microplate Reader (BMG LabTech) at excitation and emission 530/590 nm. GelTN hydrogels without HDLEC were used as a blank for the fluorescence readouts and the data were normalized accordingly.

### 2.14 Microcarrier beads sprouting assay

Cytodex3 microcarrier beads (Cytiva, Marlborough, US) were first hydrated and sterilized, as per the manufacturer's protocol, and then resuspended in EGM MV2 complete media at a concentration of 5000 beads/mL. Cytodex beads were coated with HDLEC by mixing them in a 1:400 bead to cells ratio, followed by an incubation of 4 h at 37 °C, 5 % CO_2,_ with gentle agitation every 20 min. The coated beads were then transferred to a T25 flask and incubated for up to 48 h. To embed HDLEC coated beads in GelTN hydrogel, the beads were collected and resuspended in GelTN derivatives (6 % w/v). An amount of 40 μL/well of HDLEC coated beads in GelTN hydrogel was plated in a 24 well-plate and incubated for 1.5 h for crosslinking. HDLEC coated beads were maintained in 700 μL media (with or without 100 ng/mL VEGF-C) and imaged every day for up to 5 days.

### 2.15 HDLEC spheroids generation

HDLEC spheroids were prepared using the hanging drop method [41]. 600 mg methylcellulose (4000 CP; Sigma-Aldrich) was autoclaved and dissolved in 50 mL basal EGM MV2 media at 60 °C, followed by 18 h incubation stirring at 4 °C. At confluency, HDLEC were harvested and resuspended at 40,000 cells/mL in complete EGM MV2, containing methylcellulose solution to a final concentration of 0.24 %. HDLEC were deposited in 20 μL drops onto a single well plate (Nunc™ OmniTray™). The OmniTray was then incubated upside down for 24 h (37 °C, 5 % CO_2_), allowing spheroid formation in hanging drops. HDLEC spheroids were then collected in PBS and pelleted by centrifugation. HDLEC spheroids were embedded in GelTN hydrogels (6–12 %) in a range of 15–20 spheroids/hydrogel, as previously described.

### 2.16 HDLEC sprouting assays

HDLEC spheroid-laden gelatin hydrogels were incubated in a 24-well plate (1 hydrogel/well), submerged in 700 μL EGM MV2 for 24 h at 37 °C, 5 % CO_2_. After 24 h, brightfield images were taken using an inverted microscope DMi1 (Leica Microsystems, Wetzlar, Germany) at 10X magnification. Quantification of the spheroid core area, sprout length and number was carried out using NIH ImageJ [38,39]. To assess lymphangiogenic activity in response to VEGF-C treatment, GelTN hydrogels were prepared at 6 % (GelTN_Lo) and 12 % (GelTN_Hi) using either complete EGM MV2 or serum starved (SM) media (containing 0.1 % (v/v) FCS, 0.2 μg/mL hydrocortisone, and 1 μg/mL ascorbic acid). HDLEC spheroids in SM media, were starved for 4 h before the addition of VEGF-C (in a range of 50–200 ng/mL). To incorporate VEGF-C (50 ng/mL) directly in GelTN_Hi hydrogels, VEGF-C was added to GelTN derivatives prior to cross-linking.

### 2.17 Confocal imaging

HDLEC spheroids were fixed with 4 % (w/v) paraformaldehyde (PFA; Invitrogen) for 30 min at 4 °C, and permeabilized using 0.1 % (v/v) Triton X-100 for 1 h at room temperature. The spheroids were then stained for nuclei using Hoechst 33342 (ThermoFisher Scientific), actin cytoskeleton was stained with Rhodamine Phalloidin (ThermoFisher Scientific), as per the manufacturers’ protocol. Z-stack confocal images were obtained using Leica SP8 confocal microscope at a magnification of 10X using 2 fluorescent channels to image the nucleus (blue; *λ*_exc_ = 352 and *λ*_ems_ = 455 nm) and actin filaments (red; *λ*_exc_ = 540 and *λ*_ems_ = 565 nm).

### 2.18 Reverse transcription quantitative polymerase chain reaction (RT-qPCR)

To release embedded HDLEC, GelTN hydrogels were digested using 4 mg/mL collagenase type I (Sigma Aldrich) dissolved in EGM MV2 media and incubated at 37 °C, 5 % CO_2_ for 45 min for complete digestion. Released HDLEC were collected by centrifugation. Total RNA was extracted using RNeasy kit (QIAGEN, Hilden, Germany), first strand cDNA synthesis was carried out using Superscript III Reverse Transcriptase (Invitrogen, Massachusetts, US) as per the manufacturers’ protocol. Quantitative PCR was carried out using PerfeCTa SYBR Green Fastmix (QuantaBio, Beverly MA, US) on a Bio-Rad CFX96 system. Data were analysed using the 2^-△△CT^ method [42], with the fold change being calculated in reference to the expression of the housekeeping gene *GAPDH*. Oligonucleotide sequences are shown in Supplementary Table S1.

### 2.19 Inhibition studies

For MMP inhibition, HDLEC spheroids were generated and embedded in GelTN_Lo, as previously described, then treated with 50 μM Marimastat or an equal volume of DMSO as a vehicle control. For siRNA inhibition of integrins, HDLEC were cultured in complete EGM MV2 and transfected with 20 nM siRNA against *ITGβ3* (siβ3; sc-42067, Santa Cruz) or 20 nM non-specific control siRNA (Qiagen) using RNAiMAX transfection reagent (1 μg/ml, Invitrogen). After 24 h of transfection, HDLEC were collected for either spheroid generation or RNA analysis for knockdown efficiency. The viability of HDLEC post siRNA or Marimastat treatments was assessed using AlamarBlue viability assay (Fig. S4A–B). Spheroids of siCtrl, or siβ3 treated HDLEC were then embedded in GelTN_Lo, as previously described, and treated with 50 ng/mL VEGF-C, in the presence and absence of the selective α5β1 integrin inhibitor K34C (20 μM; Biotechne, R&D systems). HDLEC spheroids were then imaged 24 h post-encapsulation to quantify sprout length and number using ImageJ.

### 2.20 VEGF-C release and biological activity

Disc shaped VEGF-C-laden GelTN hydrogels (⌀ = 6 mm) were prepared at 6 % and incubated at 37 °C, 5 % CO_2_ for 1, 3, 7, 10, 14 and 21 days in 500 μL PBS. VEGF-C diluted in PBS, prepared at the theoretical total release concentration (∼16 ng/mL) was used a control. At each time-point, GelTN hydrogels and the supernatants (500 μL/sample) were stored at −80 °C for further assessments. To quantify the released VEGF-C, a Human VEGF-C DuoSet ELISA (R&D systems) assay was performed, as per the manufacturer's protocol. To evaluate the biological activity of VEGF-C released from GelTN, HDLEC were assessed using three assays. First, a proliferation assay was conducted by seeding HDLEC in a 96-well plate at 6000 cells/well, followed by starvation in Opti-MEM I reduced serum medium (Gibco) for 18 h. Cells were then treated with either complete EGM MV2 media (CM), EGM MV2 media supplemented with 0.1 % FCS (SM), EGM MV2 media supplemented with 0.1 % FCS and 100 ng/mL VEGF-C (VC100), or supernatants obtained from the VEGF-C release experiment (GelTN_VC), including supernatant from blank GelTN. Proliferation was measured at 72 h using the AlamarBlue assay (Invitrogen). Western blot analysis was performed to assess the phosphorylation of Akt and ERK1/2 in response to VEGF-C treatment. HDLEC were seeded in 12-well plates at 150,000 cells/mL and starved for 18 h in Opti-MEM I reduced serum medium. Cells were then treated with CM, SM, VC100 or GelTN_VC for 20- and 30-min. Proteins were extracted using RIPA buffer (Sigma-Aldrich) supplemented with protease and phosphatase inhibitors (Sigma-Aldrich) and separated by polyacrylamide electrophoresis using Bolt Bis-Tris Plus Gels (Invitrogen). Proteins were transferred to PVDF membranes and incubated in blocking solution (5 % milk in PBS) for 1 h at room temperature. The membrane was then incubated with primary antibodies (diluted in 5 % BSA) against total ERK1/2 (Cell Signaling Technology, #4696T, 1:2000), phospho-ERK1/2 (Cell Signaling Technology, #4376T, 1:1000), total Akt (Cell Signaling Technology, #2920, 1:2000) or phospho-Akt (Cell Signaling Technology, #4060, 1:2000), for 18 h at 4 °C. The membrane was then washed with PBS containing 0.1 % Tween-20 (PBST, Sigma-Aldrich). This was followed by a secondary antibody incubation (diluted in 3 % milk in PBST) with either goat anti-rabbit IgG (DyLight 680) or goat anti-mouse IgG (DyLight 800, Invitrogen, 1:10000), for 1 h at room temperature. The membrane was washed with PBST and imaged using an Odyssey CLx Imaging System (LI-COR), at a scan resolution of 169 μm. The quantification of the protein band intensities was carried out using Image Studio™ Lite V5.5.4 (LI-COR) and presented as a ratio of phosphorylated protein to the total protein.

### 2.21 Subcutaneous injection

All animal experiments were conducted with ethical approval from Imperial College London in compliance with the UK Animals (Scientific Procedures) Act 1986. Female C57BL/6J mice of 8 weeks age were used for this experiment. GelTN 6 % (w/v) was prepared in 1X PBS with 64 U/mL Heparin (Sigma Aldrich) in the presence or absence of FGF-2 (400 ng/ml; Gibco). Upon mixing GelTN derivatives, the mixtures were immediately transferred into 1 mL syringes with 26G needles, followed by 20 min incubation to initiate crosslinking. Mice were sedated using 5 % isoflurane and maintained under 3 % isoflurane via a facemask during the procedure. Each mouse received two ventral subcutaneous injections of 250–300 μL GelTN solution. Post injection, the mice were routinely monitored. GelTN plugs were collected on day 14 from euthanised mice. Excised plugs were fixed in 4 % (w/v) PFA, followed by three washes with 1X PBS, and then stored in 70 % (v/v) ethanol. The plugs were sent to the Research Histology Facility at Imperial College London for paraffin embedding, sectioning (5 μm) and H&E staining.

### 2.22 Immunofluorescence staining of paraffin embedded tissue sections

The paraffin embedded tissue sections were cleared with Histological Clearing Agent (Histo-clear) for 5 min, followed by rehydration through series of ethanol concentrations (100 %, 90 %, then 70 % v/v) each for 5 min, and a final rinse in deionised distilled water (ddH_2_O). Antigen retrieval was performed using a 1 X citrate solution (Abcam). The slides in citrate solution were heated until boiling, followed by two cycles of boiling for 3 min at the lowest power. The slides were then cooled in the citrate solution at room temperature and rinsed with ddH2O. The slides were incubated in blocking buffer (3 % BSA solution prepared in PBS containing 0.05 % Tween-20) for 1 h at room temperature, followed by an incubation with anti-CD31 antibody (Abcam, #Ab28364, 1:200 dilution) or anti-FLT4 antibody (R&D Systems, #AF743, 1:100 dilution) for 18 h at 4 °C. The slides were then washed with 0.05 % PBST followed by 1 h incubation with the secondary antibody: donkey anti-rabbit IgG (Invitrogen, #A32754, 1:400 dilution) or donkey anti-goat IgG (Invitrogen, #A32816, 1:500 dilution). Both antibodies were diluted in 1 % BSA prepared in 0.025 % PBST. Imaging was carried out using a Zeiss LSM-780 inverted microscope.

### 2.23 Nucleus count of H&E images

To quantify cellular infiltration in GelTN plugs, nucleus counts were obtained from H&E-stained images. A total of 30 images were captured from various fields using Leica DM2500 upright microscope within each experimental group of GelTN plugs. The images were processed using ImageJ, specifically employing the colour deconvolution algorithm as described in Yi et al. [43]. Briefly, the images were processed using the ‘Colour Deconvolution’ function, which separates the image into two primary channels: Haematoxylin (purple) and Eosin (pink). In the Haematoxylin channel, the ‘Threshold’ was adjusted to include only the nuclei. To maintain the circularity of the nuclei and prevent merging with adjacent nuclei, the image was further processed using the ‘Watershed’ function. Nucleus counts were then quantified using the ‘Analyse Particles’ function, obtaining the number of nuclei per field and the average particle size.

### 2.24 Statistical analysis

Statistical analyses were carried out using GraphPad Prism ver.9.5. Data are presented as mean ± standard deviation (SD). The number of independent experiments is reported in the corresponding figure legends. Unpaired Student's t-test was used for comparisons between two groups; one-way ANOVA followed by Tukey's post hoc test was used to compare multiple groups; two-way ANOVA with Tukey's correction was used for experiments involving two independent variables. Significance is presented as ∗ = p < 0.05; ∗∗ = p < 0.01; ∗∗∗ = p < 0.001, and ∗∗∗∗p < 0.0001.

## 3 Results and discussion

### 3.1 Click gelatin synthesis and physical characterisation

**Fig. 1:**
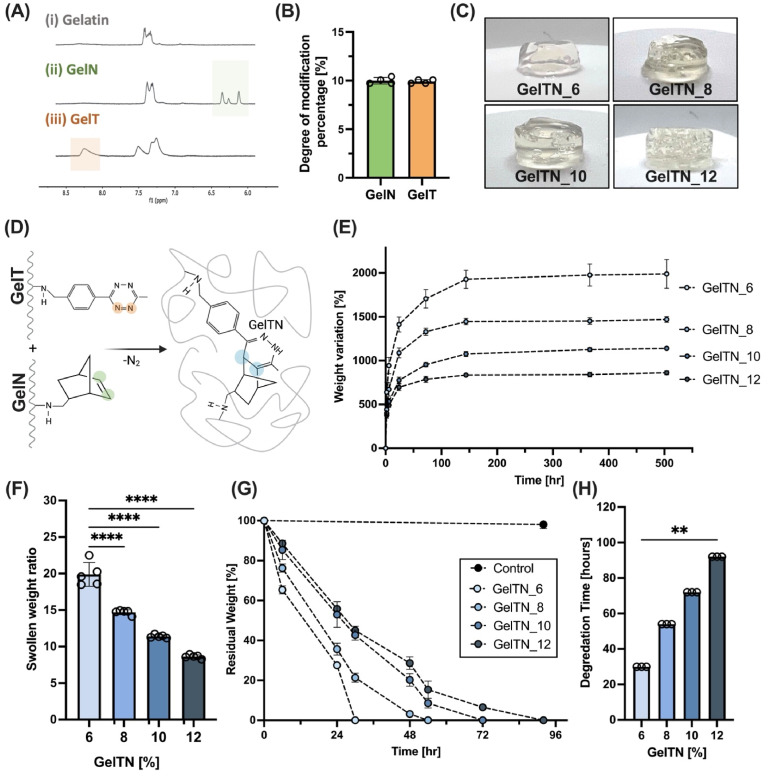
**Gelatin functionalisation with norbornene (GelN) or tetrazine (GelT) and physical characterisation**. **(A)**^1^H NMR spectra of (i) unmodified gelatin, (ii) GelN and (iii) GelT (top to bottom). **(B)** Degree of modification (DOM) percentage of GelT and GelN. **(C)** Representative images of GelTN hydrogels prepared at 6, 8, 10, and 12 % w/v (GelTN_6, GelTN_8, GelTN_10 GelTN_12, respectively). **(D)** Schematic of the bioorthogonal cross-linking between gelatin modified with tetrazine (GelT) and gelatin modified with norbornene (GelN) forming GelTN via inverse electron demand Diels-Alder reaction. **(E)** Percentage weight variation of hydrogels during swelling in PBS at 37 °C, up to 3 weeks (n = 5). **(F)** Swollen weight ratio of GelTN hydrogels in PBS at 37 °C (n = 5). **(G)** Residual weight percentage of GelTN hydrogels (6–12 %) during enzymatic degradation in collagenase type I (1 U/mL; n = 3). **(H)** Degradation time of different gelatin hydrogel concentrations (% w/v). Data are presented as mean ± standard deviation (SD; n = 3–5 per concentration).

We evaluated the chemical functionalisation of gelatin polymers and quantified the degree of modification (DOM) via proton nuclear magnetic resonance (^1^H NMR) spectroscopy was carried out. The characteristic peaks in the ^1^H NMR spectra of gelatin-tetrazine (GelT) and gelatin-norbornene (GelN) were observed at 8.5–8 ppm and 6.3–5.9 ppm, respectively (Fig. 1A)^29,32^**.** The DOM was quantified for each batch of gelatin derivatives and was found to be consistent with an average percentage of 9.9 % ± 0.2 for GelT and 9.5 % ± 0.3 for GelN (Fig. 1B). Our previous work showed that this 10.13039/100005173DOM supports cell viability, indicating its suitability as a cellular matrix for *in vitro* modelling [31,32].

To prepare click-crosslinked gelatin (GelTN) hydrogels, GelT and GelN were mixed in a 1:2 vol ratio, as previously optimised [32]. GelTN hydrogels were prepared in disc-shaped moulds at different concentrations of 6, 8, 10 and 12 % (w/v), to achieve varying gel stiffness (Fig. 1C). The hydrogel network formation occurs via an inverse electron demand Diels-Alder reaction, as illustrated in Fig. 1D. After the formation of GelTN hydrogels, samples were submerged in PBS at 37 °C to investigate their stability and swelling behaviour, represented as percentage weight variation over time (Fig. 1E). All hydrogels remained intact for 3 weeks, evidencing their capability in sustaining long-term *in vitro* cultures. A swelling equilibrium was reached after 4 days, with GelTN_6 hydrogels absorbing more water and exhibiting a higher swollen weight ratio compared to the other hydrogels prepared with higher polymer concentration (Fig. 1F). To confirm that GelTN hydrogels retain biodegradability, an enzymatic degradation assay was carried out using collagenase (1 U/mL). All GelTN hydrogels were fully degraded with the degradation kinetics varying in time, dependent on GelTN concentration (Fig. 1G). GelTN_6 hydrogels had the shortest degradation time of ∼30 h compared to GelTN_12 hydrogels, which were completely degraded by ∼92 h. Intermediate concentrations of GelTN_8 and GelTN_10 were completely degraded after 54 and 72 h, respectively (Fig. 1H).

**Fig. 2:**
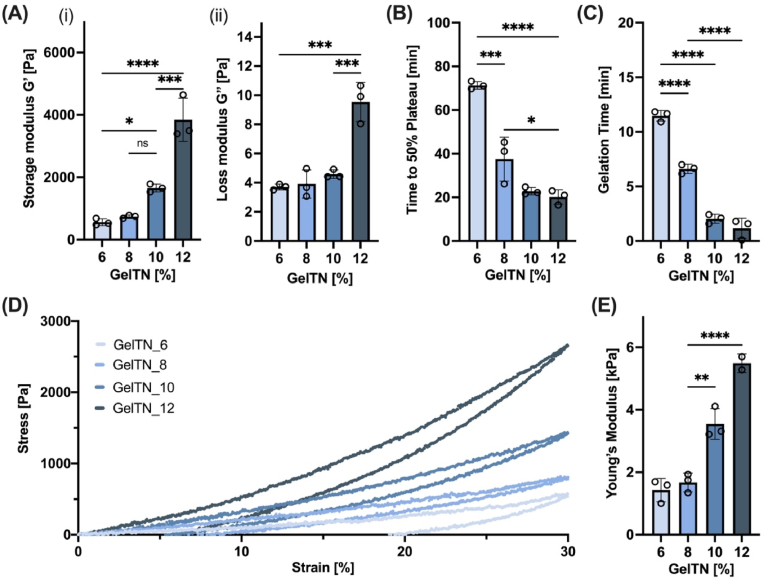
**Rheological and mechanical properties of GelTN. (A)** Average (i) storage modulus G′ and (ii) loss modulus G″ achieved at crosslinking plateau. The Average time required to reach **(B)** 50 % of G′ plateau and **(C)** the gelation of GelTN at different concentrations (6 %–12 %). **(D)** Representative stress-strain curves (compressive mechanical test) on swollen GelTN hydrogels prepared at different concentrations. **(E)** Young's modulus calculated from the linear region of the curves. Data are presented as mean ± SD (n = 3).

To assess the cross-linking kinetics of hydrogels at different GelTN concentrations (6–12 %), a rheological time sweep test was carried out to determine the storage modulus (G′) and loss modulus (G″). All GelTN concentrations have a similar G′ progression trend, showing a prompt increase after mixing gelatin derivatives before reaching a steady plateau, confirming gel formation (G’≫G″, Fig. 2A). When considering the time to 50 % of the plateau G′ value (t_50 %_ plateau), the time varied between ∼20 and 70 min, in a concentration dependent manner, with GelTN_6 showing significantly higher t_50 %_ plateau compared to GelTN_12 (Fig. 2B), indicating slower crosslinking kinetics. Moreover, the hydrogel gelation time for each concentration was determined by identifying the intersection of G′ and G’’ (tan δ = 1), which varied between ∼ 2 and 12 min, for GelTN_12 to GelTN_6, respectively (Fig. 2C). The mechanical properties of swollen GelTN hydrogels were assessed by compression tests. The stress - strain curves obtained from the compression test are shown in Fig. 2D. Hysteresis loops can be observed among all concentrations, representing energy dissipation during material deformation, a common attribute of viscoelastic materials [44]. Moreover, the maximum stress value was found to increase in a concentration-dependent manner (∼500–2500 Pa). Accordingly, Young's modulus was found to vary between ∼1.4 and 5.4 kPa (Fig. 2E), which falls within the range of the elastic modulus of the vascular endothelium [45].

### 3.2 Cytocompatibility of GelTN for culturing lymphatic endothelial cells

HDLEC were top seeded onto GelTN hydrogels (6–12 %) to assess cell adhesion on the 2D hydrogel surface. HDLEC adhered to GelTN hydrogels at all concentrations and achieved confluence after 72 h of culture (Fig. S1A). HDLEC top-seeded onto GelTN_12 exhibited significantly larger cell area compared to those seeded on hydrogels prepared at lower concentrations (Fig. S1B). HDLEC cultured on lower concentrations of GelTN (<10 % w/v), had an average area of 1000–1100 μm^2^, whereas, on GelTN_12, HDLEC had an average area of ∼1500 μm^2^ (Fig. S1B). This response suggests that as the stiffness of GelTN hydrogels increases, HDLEC spread more extensively, covering a larger hydrogel surface area.

**Fig. 3:**
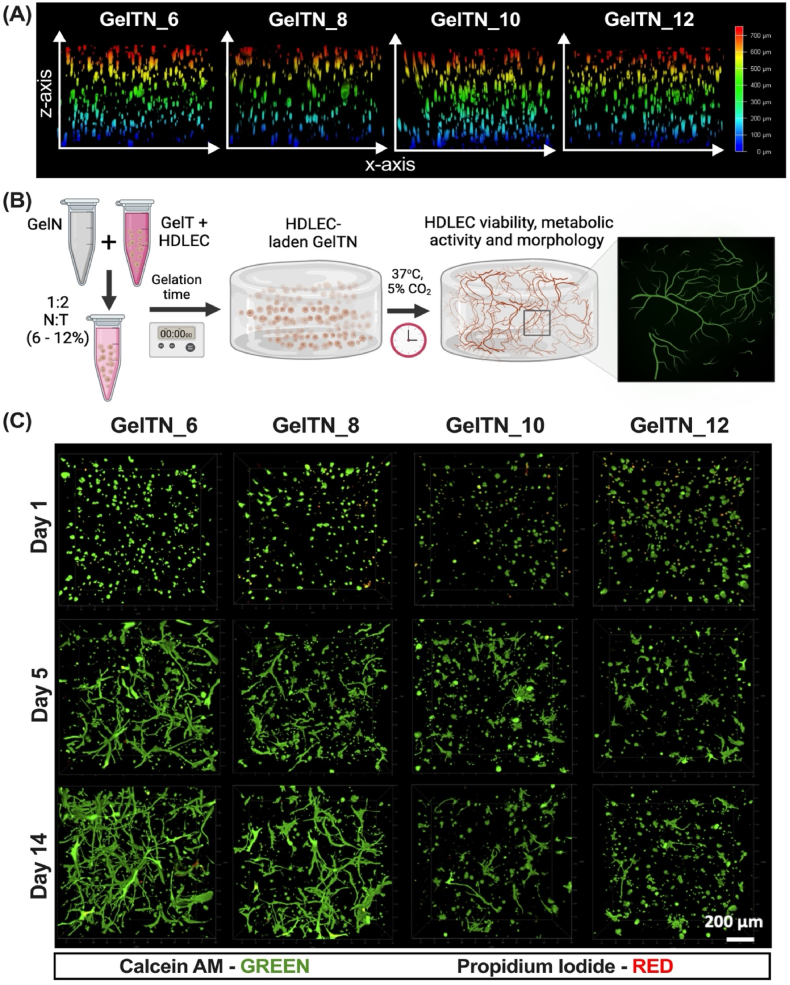
**Three-dimensional (3D) distribution of HDLEC within GelTN hydrogels. (A)** Orthogonal section view of Z-stack confocal images (700 μm) of HDLEC encapsulated in GelTN (6–12 % w/v), stained with calcein-AM, presented with depth coding to indicate the z-position (depth) of HDLEC within GelTN network. The scale bar indicates the depth range of 0–700 μm, as a reference for the z-axis positioning of HDLEC within GelTN hydrogels. **(B)** Schematic illustration of HDLEC encapsulation in GelTN hydrogel, followed by compatibility assessments. **(C)** Top view (X – Y plane) of z-stack confocal images of HDLEC embedded in GelTN (6–12 %) hydrogels for up to 14 days, stained with calcein AM (green) and propidium iodide (red). Scale bar = 200 μm.

To achieve an optimal 3D distribution of HDLEC in different focal planes within the GelTN network, the mixture of cells and GelTN were incubated until the hydrogel reached the gelation time, as determined by the rheological test, before hydrogels were cast into the disc-shaped moulds. As presented in z-stack images in Fig. 3A, this approach resulted in an even distribution of HDLEC across the z-plane at all GelTN hydrogel concentrations. In contrast, achieving uniform distribution of cells by encapsulated them in other matrices (e.g. fibrin) is reported to be challenging, as the rapid gelation time results in a less controlled and often inconsistent distribution of cells [46,47]. Furthermore, the high specificity of the bioorthogonal click reaction allows the encapsulation of HDLEC without interfering with biological processes, as per bioorthogonal reaction definition [28,32]. Importantly, when calculating the Young's modulus and assessing the rheological properties for fresh and swollen GelTN hydrogels, no significant impact was observed on the initial mechanical properties of GelTN (Fig. 2SA).

**Fig. 4:**
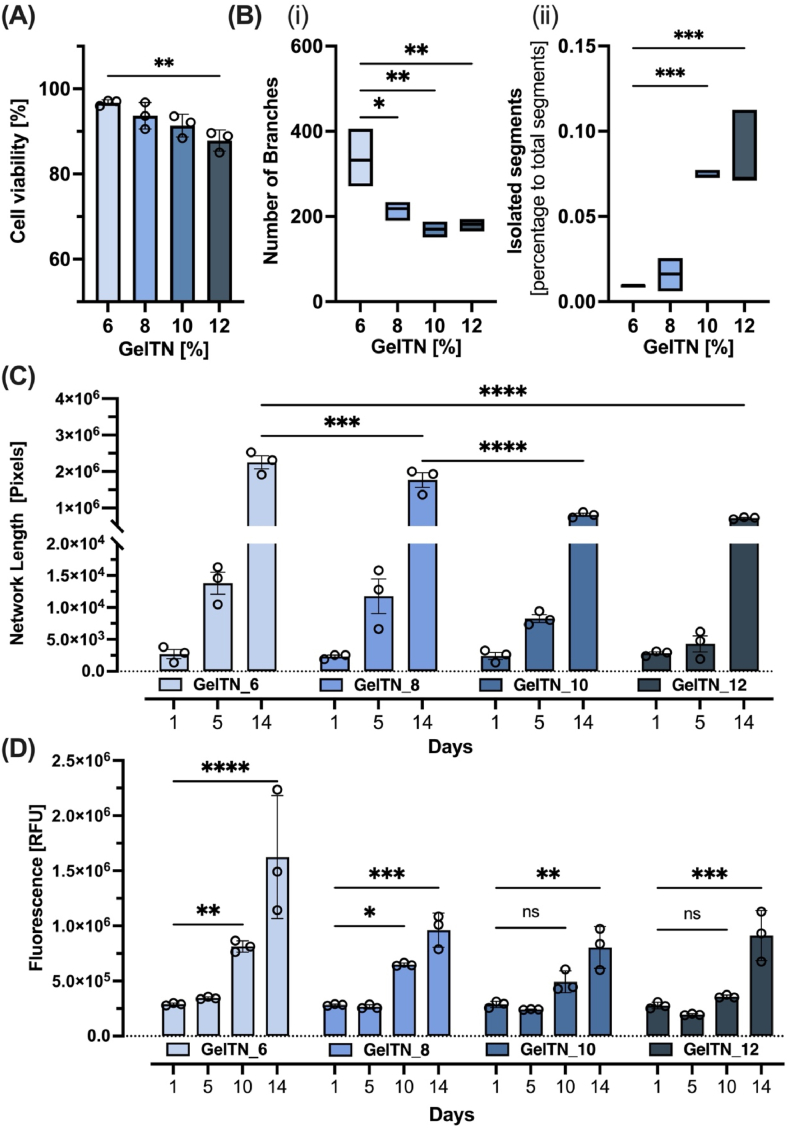
**Quantification of HDLEC network formation, viability and metabolic activity in response to 3D encapsulation in GelTN. (A)** Percentage of viable cells after 24 h of encapsulation. **(B)** Quantification of (i) branches, (ii) isolated elements and **(C)** total network formation of HDLEC-laden in GelTN hydrogels (6–12 %) after 14 days. **(D)** Metabolic activity of HDLEC encapsulated in GelTN (6–12 %) at day 1, 5, 10 and 14, presented as relative fluorescence unit (RFU) of AlamarBlue. Data are presented as mean ± SD (n = 3).

To evaluate GelTN cytocompatibility, embedded HDLEC were cultured for 14 days and assessed for their cellular viability and metabolic activity (Fig. 3B). Fig. 3C shows maximum projection images from confocal z-stacks revealing the 3D distribution of HDLEC over time in GelTN hydrogels. On day 1, HDLEC maintained a circular morphology in all GelTN concentrations. When quantifying viable cells, the percentage viability of HDLEC was significantly reduced in GelTN_12, nevertheless, the percentage viability still surpassed 85 % for all the GelTN formulations (Fig. 4A). Overtime, GelTN hydrogels supported self-assembled HDLEC network formation in a concentration dependent manner. By day 5, significantly more elongated lymphatic networks were observed in GelTN_6 and GelTN_8 hydrogels, compared to the other formulations (Fig. 3, Fig. 4B – C). The metabolic activity of HDLEC embedded in all GelTN hydrogels were not significantly different from one another up to day 5. By day 10, HDLEC metabolic activity increased significantly for GelTN_6 and GelTN_8, but not for GelTN_12 (Fig. 4D). However, all GelTN concentrations achieved a significant increase in HDLEC metabolic activity by day 14, with the cellular metabolic activity in GelTN_6 being significantly higher compared to other concentrations.

### 3.3 GelTN hydrogels with softer elastic modulus supports HDLEC spheroid sprouting for lymphangiogenesis modelling

**Fig. 5:**
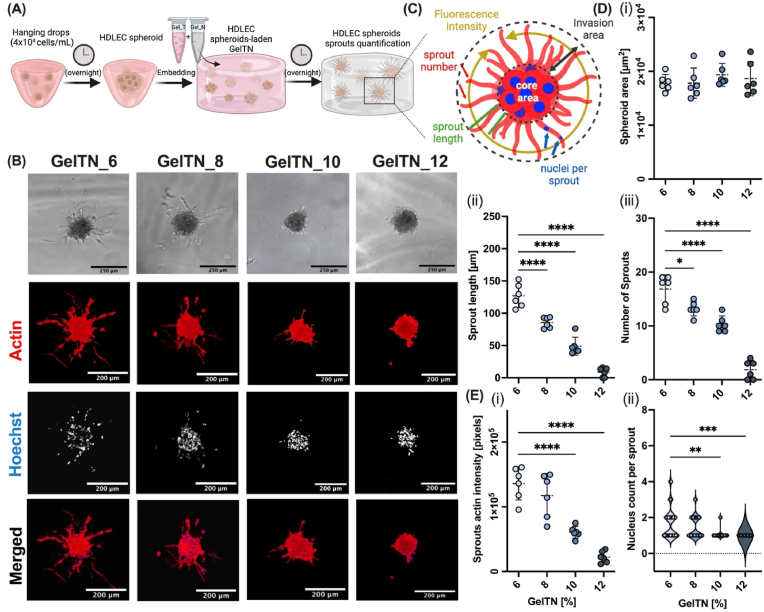
**Sprouting of HDLEC spheroids in GelTN at different concentrations after 24 h of encapsulation**. **(A)** Schematic illustration of HDLEC spheroid generation and encapsulation using hanging drop method, followed by spheroid encapsulation in GelTN. **(B)** Representative brightfield (BF) images of HDLEC spheroids (scale bar = 250 μm) and z-stack confocal images (projection of x – y planes) of HDLEC spheroids stained with Hoechst (Blue) and Phalloidin (actin filaments, red). Scale bar = 200 μm. **(C)** Schematic illustration of the quantification method of HDLEC spheroid sprouts. **(D)** The quantification of HDLEC spheroids (i) sprout length and (ii) number. (**E)** Quantification of (i) actin intensity and (ii) nuclei count per sprout. The quantification was carried out on 6 random spheroids for each experimental group. Data are presented as mean ± SD (n = 3).

Endothelial cell spheroids are cell aggregates that can give rise to cell sprouts with a capillary-like phenotype when grown in a permissive ECM, which can be used to assess angiogenic activity [41]. The incorporation of HDLEC spheroids in GelTN enables the creation of a 3D cell culture platform that more closely mimics the biomechanical cell-matrix interactions during lymphangiogenesis [33,41]. HDLEC spheroids were generated using the hanging drop method and encapsulated in different concentrations of GelTN (Fig. 5A). Within 24 h after encapsulation, capillary-like sprouts emerged from HDLEC spheroids in a GelTN concentration dependent manner. Higher concentrations of GelTN appeared to inhibit sprout formation (Fig. 5B). At all GelTN concentrations, HDLEC spheroid core size remained consistent, with an average core area ranging between 17,809 and 19,341 μm^2^ (Fig. S2B). In GelTN_6, the average number of HDLEC sprouts was ∼17 sprouts per spheroid, with an average sprout length of 127 μm (Fig. 5D). Whereas, in GelTN_12, the average sprout number was significantly reduced to ∼2 sprouts per spheroid, with an average sprout length of 8.6 μm (Fig. 5D). These data are in line with a significant decrease in nucleus count per sprout and actin staining intensity at concentrations >8 % GelTN (Fig. 5E).

**Fig. 6:**
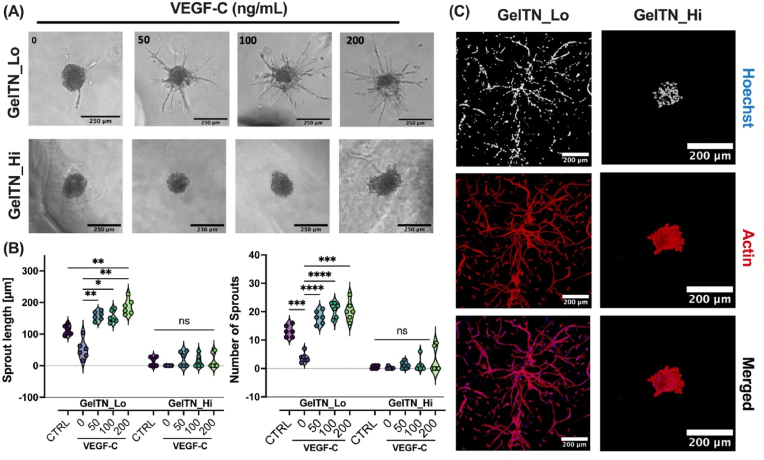
**Sprouting of HDLEC spheroids encapsulated in GelTN_Lo (6 % w/v) and GelTN_Hi (12 % w/v) hydrogels in response to vascular endothelial growth factor C (VEGF-C). (A)** BF images of HDLEC spheroids embedded in GelTN_Lo and GelTN_Hi, supplemented with complete media (CTRL), or serum starved media (0.1 % FCS) with different concentrations of VEGF-C (0, 50, 100, and 200 ng/mL). **(B)** Quantification of HDLEC spheroids sprout length (left) and number (right) was calculated for 6 randomly selected spheroids per experimental group. **(C)** Representative confocal images of HDLEC after 72 h of hydrogel encapsulation (nuclei = blue, actin = red; scale bar = 200 μm).

To assess the responsiveness of HDLEC-laden GelTN hydrogels to growth factor stimulation, a range of 0–200 ng/mL of VEGF-C was added to GelTN_6 (hereafter referred to as GelTN_Lo) and GelTN_12 (referred to as GelTN_Hi). After 24 h, HDLEC spheroids encapsulated in GelTN_Lo showed VEGF-C dependent increases in sprout length and number compared to the GelTN_Lo without VEGF-C (Fig. 6A and B). In contrast, HDLEC spheroids embedded in GelTN_Hi were unresponsive to VEGF-C at any concentration (Fig. 6A and B). Interestingly, prolonging VEGF-C treatment up to 72 h promoted sprout expansion and cell migration away from the spheroid core in GelTN_Lo (Fig. 6C, Fig. S2C). However, this was not observed in GelTN_Hi where HDLEC sprouting remained absent even after 72 h (Fig. 6C, Fig. S2C). The use of cell spheroids overcomes several technical challenges encountered with commonly used microcarrier beads. These include (1) achieving optimal cell attachment and growth, (2) mechanical stress and shear forces of microcarrier bioreactors, (3) batch-to-batch variability, (4) sedimentation leading to inconsistent cell distribution, and (5) time inefficiency, as cell sprouts take at least 3–5 days to be observed using the bead assay (Fig. S3) [48,49]. These technical challenges can be easily overcome using HDLEC spheroids culture in our hydrogels, as we obtained reproducible and consistent sprouting in just 24 h of encapsulation in GelTN. Moreover, supplementation with VEGF-C promoted HDLEC spheroids to form a lymphatic cell network in soft hydrogels (GelTN_Lo) within 72 h. Having established a 3D *in vitro* sprouting model of lymphangiogenesis, we then explored the impact of GelTN hydrogel's physico-mechanical properties on HDLEC sprouting. By shifting the focus from model development to the subsequent investigation of hydrogel properties, we bridge a crucial gap in understanding the interplay between mechanical cues and lymphangiogenesis [50].

**Fig. 7:**
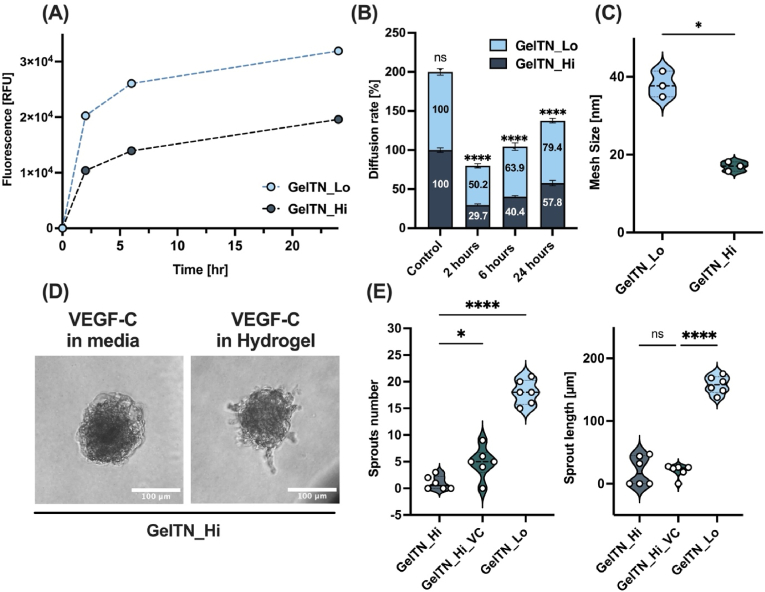
**The effect of GelTN concentration on the hydrogel diffusion rate and mesh size and HDLEC on sprouting.** GelTN_Lo and GelTN_Hi were prepared and incubated in 2 mg/mL FITC-Dextran (20 kDa) for up to 24 h with the **(A)** fluorescence measured post-digestion after 0, 2, 6 and 24 h, and **(B)** the percentage diffusion rate was calculated using the control hydrogel (containing 2 mg/mL FITC-Dextran). Statistical analysis represents the significant difference of diffusion rate between GelTN_Lo and GelTN_Hi. **(C)** Estimated mesh size of GelTN_Lo and GelTN_Hi (n = 3). **(D)** Bright field images of HDLEC spheroids encapsulated in: (Left) GelTN_Hi immersed in media containing 50 ng/mL VEGF-C, or (right) GelTN_Hi containing 50 ng/mL VEGF-C (GelTN_Hi_VC) for 24 h (scale bar = 100 μm). **(E)** The quantification of HDLEC sprout (i) number and (ii) length, embedded in GelTN_Hi and GelTN_Hi_VC compared to GelTN_Lo for 24 h.

To examine whether the different phenotypes of HDLEC spheroids encapsulated in GelTN_Lo or GelTN_Hi was due to differences in the diffusion rates of growth factors, GelTN hydrogels were immersed in fluorescein isothiocyanate-Dextran (FITC-Dex) with a molecular weight similar to human recombinant VEGF-C (∼20 kDa) [51,52] and incubated at different timepoints. GelTN_Lo and GelTN_Hi showed significantly different FITC-Dex diffusion rates in a time-dependent manner (Fig. 7A and B). GelTN_Lo achieved ∼50 % release of FITC-Dex, whereas GelTN_Hi achieved ∼30 % release, by 24 h (Fig. 7B). These data suggests that hydrogel mesh size might affect VEGF-C diffusion and impose a physical constraint that influences cellular physiology [[53], [54], [55]]. When calculating GelTN hydrogels mesh size, significant differences between GelTN_Lo (∼40 nm) and GelTN_Hi (∼20 nm) were observed (Fig. 7C). However, given that the estimated hydrodynamic radius of VEGF-C (2–3 nm) [[56], [57], [58]] is considerably smaller than both GelTN mesh sizes, it is unlikely that VEGF-C diffusion alone accounts for the observed reduction in HDLEC sprouting in GelTN_Hi. Instead, other factors, such as the mechanical properties of the hydrogel and the physical constraints it imparts on cellular behaviour, may be more influential in regulating sprouting. To further investigate this, VEGF-C (50 ng/mL) was directly embedded in GelTN hydrogels upon preparation, rather than being added to the culture medium (Fig. 7D). While this approach marginally increased HDLEC sprouting in GelTN_Hi, encapsulated spheroids still sprouted less compared to those in GelTN_Lo (Fig. 7E). This highlights the possibility that hydrogel stiffness, independent of diffusion constraints, plays a critical role in regulating lymphangiogenesis. While analysis of the physico-mechanical properties of GelTN hydrogels lays the foundation for understanding lymphangiogenesis *in vitro*, we further explored intracellular signalling pathways is required to clarify the interplay between matrix mechanics and cellular responses.

### 3.4 MMP-mediated degradation of GelTN and integrin-dependent cell matrix interactions promote sprouting of HDLEC in GelTN hydrogels

Previous studies conducted in 2D environments have established that substrate stiffness significantly influences LEC behaviour, including migration, proliferation, and tube formation. For instance, Alderfer et al. demonstrated stiffness-dependent modulation of lymphatic tube formation and VEGF-C responsiveness using hyaluronic acid (HA) hydrogels [59]. Similarly, Qin et al. reported enhanced lymphangiogenesis driven by matrix stiffness using gelatin methacryloyl (GelMA) hydrogels, and linked this response to increased activation of FAK signalling pathways [16]. Furthermore, Frye et al. demonstrated that transcriptome analysis of LEC grown on soft matrices revealed increased GATA2 expression, which in turn drove the upregulation of genes involved in cell migration and lymphangiogenesis, including *VEGFR3* [60]. However, these findings are predominantly derived from 2D assays, which do not fully recapitulate the complex, three-dimensional cell–ECM interactions encountered *in vivo*. By employing GelTN hydrogels, our study advances beyond the previous 2D approaches, providing a more physiologically relevant 3D context to investigate how matrix stiffness influences lymphatic sprouting.

**Fig. 8:**
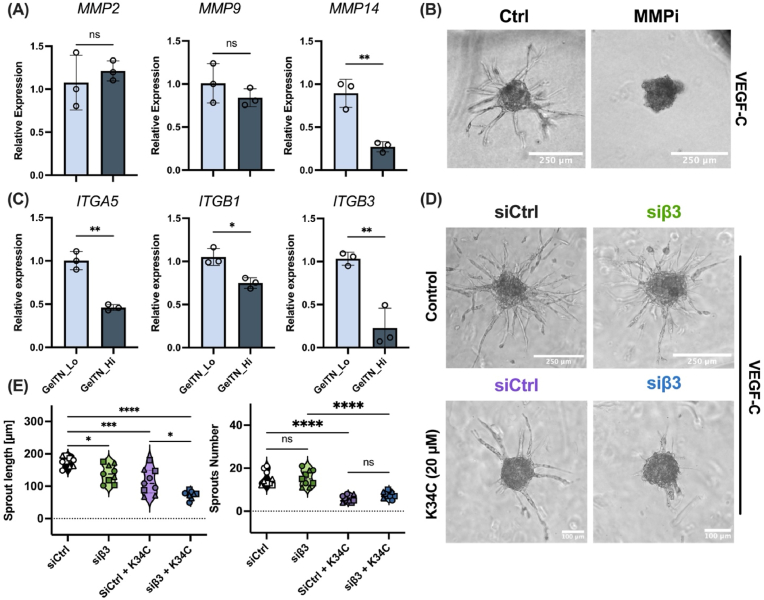
**Mechanical regulation of HDLEC lymphangiogenic activity in GelTN_Lo and GelTN_Hi via matrix metalloproteases (MMPs) and integrins. (A)** RT-qPCR of the relative expression of (i) *MMP2*, *MMP9* and *MMP14* in HDLEC encapsulated in GelTN_Lo vs GelTN_Hi (n = 3; scale bar = 250 μm). **(B)** Brightfield images of HDLEC spheroids 24 h post embedding in GelTN_Lo supplemented with 50 ng/mL VEGF-C in the presence or absence of 50 μM Marimastat (MMPi). **(C)** Relative expression of α5 (*ITGα5*), β1 (*ITGβ1*), and β3 (*ITGβ3*) integrins in HDLEC encapsulated in 1GelTN_Lo vs GelTN_Hi (n = 3). **(D)** Brightfield images of siCtrl and siβ3 transfected HDLEC spheroids encapsulated in GelTN_Lo, cultured in 50 ng/mL VEGF-C containing media (i and ii), or 50 ng/mL VEGF-C containing media with 20 μM K34C (iii and iv). Scale bar = 100–250 μm. **(E)** Quantification of HDLEC spheroid sprouts length (left) and number (right) was calculated for 9 randomly selected spheroids per experimental group. Data are presented as mean ± SD.

Matrix metalloproteases belong to the family of proteolytic enzymes that contain Zn^2+^ and Ca^2+^ ions in their active site [61]. Among different types of MMPs, MMP2 (gelatinase-A) and MMP9 (gelatinase-B) are reported to induce the release of VEGF upon ECM degradation and promote angiogenesis in ECs [62,63]. In addition, membrane type-1 (MT-1) MMP, also known as MMP14, is a key regulator of ECM degradation, and is directly associated with the VEGF-C/VEGFR3 signalling pathway and lymphangiogenesis [64]. We showed that the expression of MMP14 is dependent on hydrogel stiffness, with *MMP1*4 mRNA levels significantly downregulated in HDLEC embedded in GelTN_Lo compared to GelTN_Hi hydrogels (Fig. 8A). However, expression of *MMP2* and *MMP9* were not significantly affected (Fig. 8A). To investigate the role of MMP-mediated gelatin degradation during HDLEC sprouting, HDLEC spheroids were cultured in GelTN_Lo in the presence of Marimastat, an MMP-inhibitor [51,52], or DMSO as a vehicle control. As shown in Fig. 4SC, DMSO treatment did not significantly affect sprouting compared to the untreated control. In contrast, HDLEC spheroids failed to sprout in the presence of Marimastat, even after VEGF-C treatment (Fig. 8B). This highlights the importance of MMP-mediated ECM remodelling and degradation to facilitate HDLEC sprouting in permissive GelTN hydrogels.

In addition to MMPs, integrin cell surface receptors play an important role in coupling the actin cytoskeleton to the ECM [17]. This serves as a mechanical anchoring mechanism between the cell and the surrounding matrix, and activates the signalling pathways downstream of integrins [17]. The integrin family consists of two subunits, alpha (α) and beta (β), forming different combinations of heterodimers that are unique to particular cell types and extracellular environments [65,66]. There are different subsets of integrin receptors that are specifically associated with collagen binding. Of particular relevance to endothelial cells are those integrins, such as α5β1 and αVβ3, that recognise the RGD motif present in gelatin [66,67].

We therefore focussed on expression of these integrin subunits in response to GelTN HDLEC encapsulation. A significant down-regulation in the mRNA expression of these integrin subunits was detected in GelTN_Hi compared to GelTN_Lo hydrogels (Fig. 8C, Fig. S4D). This may be due to the reduction of traction forces imposed by encapsulated HDLEC in GelTN_Hi, given that the GelTN network is composed of stable, non-dynamic covalent crosslinks. The stress-relaxation behaviour of hydrogels have been previously reported to play a critical a role on β1 integrin localisation and activity [68]. When comparing the stress-relaxation profiles of GelTN hydrogels in this study, we found that the GelTN_Hi hydrogel relaxation rate was ∼2.39 × 10^6^ times slower than GelTN_Lo (Fig. S4E). This was also reported by Wei et al., who showed that human endothelial colony forming cells (ECFC) encapsulated in elastic hydrogels are unable to contract, resulting in reduced integrin clustering, activation and expression [17]. Although it is still debated whether integrin clustering and activation are stimulated via outside-in or inside-out signalling [17], studies suggest the involvement of focal adhesion kinase (FAK) in cell contractility and promotion of integrin clustering and signalling propagation in an outside-in manner [17,69]. In our study, the integrin-dependent sprouting of HDLEC was shown through inhibition and knockdown studies.

To better understand the role of integrins in regulating HDLEC sprouting in the GelTN_Lo hydrogels, the expression of ITGβ3 in HDLEC was inhibited by siRNA (siβ3; Fig. S4F), in the presence or absence of the α5β1 inhibitor K34C. Although the knockdown of ITGβ3 did not influence the number of HDLEC sprouts, their length was significantly decreased compared to the siCtrl-treated cells (Fig. 8D and E). Inhibition of α5β1 had a more pronounced effect on HDLEC sprouting; treatment with K34C reduced both the number and length of the HDLEC sprouts (Fig. 8D and E). Interestingly, co-inhibition of ITGβ3 and α5β1 appeared to show a synergistic effect in reducing HDLEC sprout length (Fig. 8E). This may suggest a synergistic interaction between these integrins in controlling HDLEC-ECM interactions and lymphangiogenesis [70], where endothelial cells actively invade and extend through the matrix network, which is not captured in traditional 2D models. In fact, it is important to note that integrin activation is context-dependent and differs substantially in 3D versus 2D environments [71,72]. In 2D cultures, integrins primarily mediate cell adhesion to a flat substrate, whereas in 3D matrices, integrins also regulate migration through dynamic ligand interactions, contractility, and matrix remodelling [71,72].

While the knockdown of β1 integrin is known to suppress the migration of HDLEC and inhibit *VEGFR3* [70], the detailed role of β3 integrin in lymphangiogenesis is yet to be explored. In a previous study by Landau et al.*,* bulk RNA-Seq data revealed an enrichment in the β3 integrin cell surface interaction pathway in a 3D co-culture of HDLEC and dental pulp stem cells (DPSCs) [73]. The co-culture of HDLEC with DPSCs was reported to enhance lymphatic vessel formation, nevertheless, the role of integrin β3 was not further investigated [73]. Herein, our data reveal that β3 integrin contributes to HDELC sprouting in 3D GelTN hydrogels. Previous work by Driscoll et al., has established integrins not only as adhesive molecules but also as true mechanosensors capable of transducing extracellular mechanical cues into intracellular biochemical signals [74]. Notably, αVβ3 integrin has been shown to regulate downstream mechanosensitive pathways such as FAK and YAP/TAZ in a force- and stiffness-dependent manner in mouse lung endothelial cells [74]. This is particularly relevant, given the stiffness range of our GelTN hydrogels (1.4–5.4 kPa), which aligns with the stiffness window shown to trigger integrin conformational activation and subsequent downstream signalling in endothelial cells [74]. Further studies are needed to investigate whether β3 integrin regulates HDLEC sprouting through stiffness-dependent activation of downstream pathways such as FAK and RhoA.

### 3.5 Testing GelTN injectability for growth factor delivery *in vivo*

Therapeutic lymphangiogenesis is a promising strategy for promoting the growth of lymphatic vessels in pathological conditions, such as lymphoedema [75], myocardial infarction [76], and wound healing [77], to **restore the lymphatic system's vital role in fluid balance, immune cell trafficking, and tissue regeneration**[78]**.** One approach to achieving therapeutic lymphangiogenesis is the overexpression of VEGF-C using adenoviral vectors or administration of recombinant VEGF-C [79,80]. However, these methods face challenges in achieving controlled delivery due to the rapid degradation and short half-life of VEGF-C *in vivo* [81,82]. Thus, delivery of VEGF-C often necessitates high doses or repeated administration, potentially leading to off-target effects and increased costs, highlighting the need for a localised and sustained delivery system [83]. Compared to cell-laden hydrogels, growth factor-based systems offer a cell-free alternative that can overcome challenges related to immunogenicity, viability, and trafficking hurdles, while still promoting endogenous lymphangiogenesis [84,85]. Biomaterial-mediated delivery of growth factors has emerged as a promising tool for achieving therapeutic lymphangiogenesis through the localised and sustained delivery of VEGF-C^83^. The use of click chemistry to modify gelatin polymers offers additional advantages, including providing tunable mesh sizes, tailored physico-mechanical properties, and enhanced injectability [30]. Therefore, beyond their use in 3D *in vitro* models, we characterised GelTN hydrogels for their injectability and ability to mediate sustained release of VEGF-C, while also preserving its biological activity for driving *in vivo* lymphangiogenesis.

**Fig. 9:**
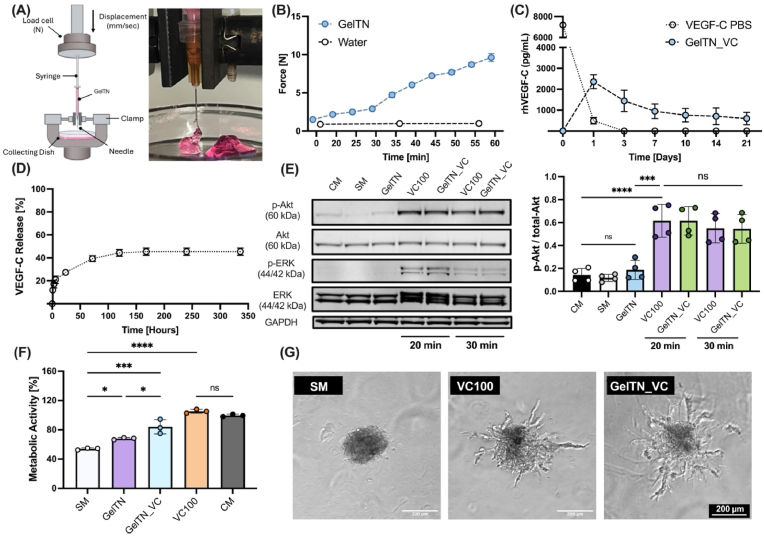
**Evaluation of GelTN hydrogel injectability and its potential as a growth factor delivery platform. (A)** Schematic illustration of the mechanical test setup to measure the injection force of GelTN primed in 1 mL syringe and 26G needle. **(B)** Representative Force – Time plots of GelTN (6 % w/v), ejected at 0 then every 5 min at 20 min post-crosslinking up to 60 min (n = 5). Water was used as a control. **(C)** VEGF-C release from GelTN in comparison to VEGF-C in PBS (control; n = 3). **(D)** Percentage VEGF-C release determined by cumulative VEGF-C concentration divided by the initial total VEGF-C concentration, multiplied by 100 (n = 3). **(E)** Representative images of western blot analysis showing p-Akt (60 kDa) and p-ERK1/2 (44 and 42 kDA) levels in HDLEC treated with GelTN_VC, V_100, CM, SM and GF-free GelTN (NC), for 20- and 30-min. Total Akt and ERK1/2 levels measured in comparison to the phosphorylation levels. GAPDH (36 kDa) was used as a loading control (Left panel). Quantification of p-Akt/total-Akt ratio in HDLEC treated with supernatants from SM, GelTN, GelTN_VC, VC100 and CM, for 20 and 30 min (right panel, n = 4). **(F)** Metabolic activity of HDLEC after treatment with supernatants from GelTN_V hydrogels, GelTN hydrogels, and controls: CM, SM and 100 ng/mL VEGF-C (V_100) for 72 h. Percentage HDLEC metabolic activity relative to the SM control (n = 3). **(G)** Representative brightfield images of HDLEC spheroids encapsulated in GelTN and treated with SM, VC100 and GelTN_VC for 24 h (Scale bar = 200 μm). Data are presented as mean ± SD.

To assess the injectability of GelTN for subcutaneous administration, the extrusion of GelTN_Lo was first assessed qualitatively using a 1 mL syringe fitted with a 26G needle, at different time points ranging from 20 to 96 min post-mixing of GelT and GelN derivates. As shown in Fig. S5, the hydrogel began forming continuous GelTN filaments approximately 26 min after crosslinking. The images indicate that GelTN hydrogels maintained smooth and continuous extrusion up to approximately 64 min post-crosslinking. Beyond this time, the GelTN filaments exhibited increasing irregularities and fragmentation. To assess GelTN injectability quantitively, the force required for GelTN ejection was measured at a speed simulating the subcutaneous injection rate of 1 mL/11 sec^37^ (Fig. 9A). The force required for extrusion of GelTN increased steadily, with values ranging from around 2 N at 20 min to approximately 10 N at 60 min (Fig. 9B). According to the international organisation of standardisation (ISO), guidance 11608-3, section 4.3, the maximum force for subcutaneous injection should not exceed 20 N for a flow rate of 1 mL/sec^37^, suggesting that GelTN is within the acceptable range for subcutaneous injection. Notably, the gelation of GelTN was observed to be slow enough to prevent needle blocking, but also quick enough to prevent the dispersion of the gel precursor solution from the injected site. Based on the quantitative and qualitative analysis of GelTN injectability, the time-window for injection was determined to range between 20 and 60 min post-crosslinking (Fig. 9B). This allows for greater flexibility in clinical settings for preparation and handling prior to injection. In contrast, injectable hydrogels with fast gelation times can pose several practical challenges [86,87]. For instance, alginate hydrogels, while demonstrated to sustain the release of VEGFs, are known to gel rapidly upon contact with ionic crosslinking agents (e.g. Ca^2+^), often within seconds [[88], [89], [90]]. This rapid gelation can lead to inconsistent gel formation, potentially compromising the homogeneity of the drug distribution and efficacy [86]. Additionally, the short handling time necessitates quick injection, which can be technically challenging and increase the risk of complications during the procedures [91].

Here, we showed that the release kinetics of VEGF-C allowed the ability of GelTN to sustain the release of VEGF-C over a period of 3 weeks, compared to the carrier-free control VEGF-C which degraded promptly after 24 h of incubation (Fig. 9C). Additionally, the cumulative release profile of VEGF-C from GelTN showed a gradual increase, reaching a plateaued release of ∼45 % after 5 days following VEGF-C encapsulation (Fig. 9D).

When engineering hydrogel-based delivery systems, maintaining the bioactivity of the encapsulated molecule can pose a significant challenge [92]. To confirm that the bioorthogonal crosslinking used in GelTN hydrogels does not compromise the bioactivity of the encapsulated VEGF-C, we assessed its biological activity through various functional and molecular assays.

The stimulation of HDLEC with VEGF-C is well-known to result in rapid activation of both MAPK/ERK and PI3K/Akt signalling pathways [93]. Herein, the treatment of HDLEC with VEGF-C released from GelTN (GelTN_VC) resulted in a significant phosphorylation of both Akt and ERK (Fig. 9E and Fig. S6), confirming that released VEGF-C is functionally active. Moreover, the treatment of HDLEC with GelTN_VC was shown to significantly increase HDLEC metabolic activity after 72 h of treatment (Fig. 9F). Interestingly, the metabolic activity of HDLEC treated with GelTN control supernatant was also increased compared to the serum staved media (SM). To further assess the sustained long-term bioactivity of released VEGF-C, supernatants collected after up to 10 days of release were used to treat HDLEC, which continued to promote a significant increase in metabolic activity compared to the SM control (Fig. S7). This suggests the added benefit of using gelatin as a backbone polymer, not only for its physical properties but also for its pro-proliferative effects, as previously described by Mogha et al. [94]. To further assess the bioactivity of the supernatant containing released VEGF-C, HDLEC sprouting assay was carried out using conditioned media from VEGF-C-containing GelTN. As demonstrated in Fig. 9G the treatment of HDLEC spheroids with GelTN_VC promoted their sprouting, confirming that GelTN preserves VEGF-C bioactivity.

### 3.6 Subcutaneous injection of GelTN *in vivo*

**Fig. 10:**
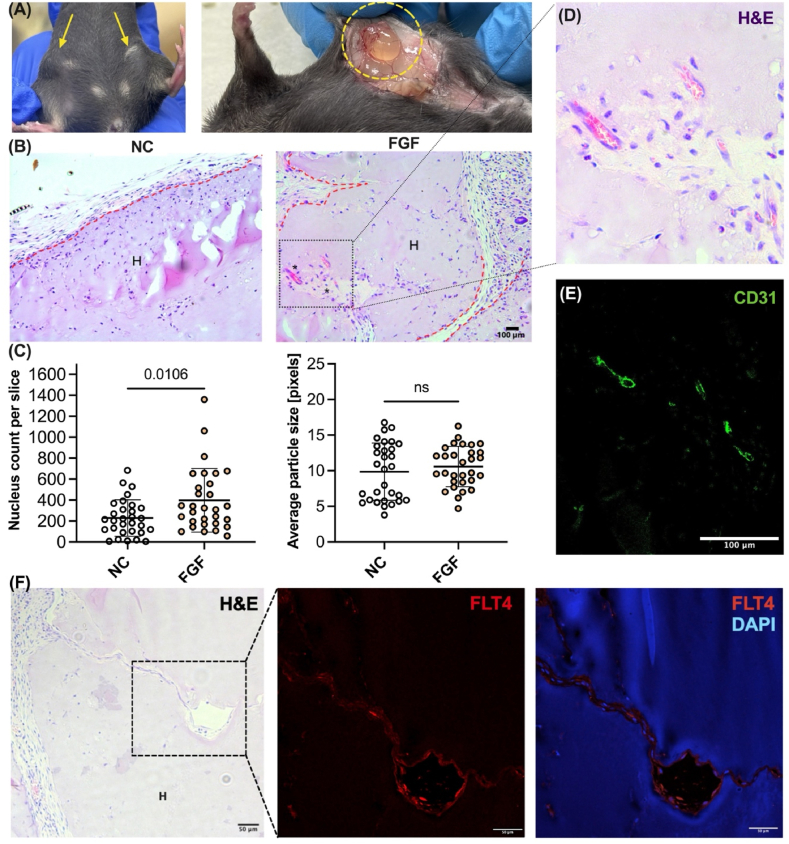
**Cell infiltration and CD31^+^ neo-vessel formation in subcutaneously injected GelTN. (A)** Snapshot images of the ventral view of C57BL/6J mouse subcutaneously injected with two GelTN hydrogels (yellow arrow; left). Closer view of the injection site after dissection, showing the injected GelTN hydrogel plug collected at 2 weeks post-injection (yellow circle; right). **(B)** Representative brightfield images (10X) showing sections of blank GelTN (NC) and FGF-laden GelTN. The red dashed lines indicate the boundary between hydrogel plug (H) and mouse tissue. **(C)** Number of haematoxylin-stained nuclei (left) and average size (right) were quantified for cell infiltration at different captured fields (n = 4 plugs, 30 analysed fields). **(D)** Perfused vessels in FGF-laden GelTN hydrogel highlighted in the magnified inset from panel B. **(E)** IF images of CD31 positive staining of the perfused vessels detected in panel D. **(F)** Representative haematoxylin and Eosin image of perfused LEC in FGF-laden GelTN hydrogel plug (H; left). Immunofluorescence staining for FLT4 (VEGFR3; Red) reveals positive lymphatic endothelial cell signal (Middle). Merged image showing FLT4 (red) and DAPI (blue), confirming lymphatic endothelial cell localisation within the hydrogel (Right). DAPI signal was detected at 358 nm, where gelatin exhibits mild autofluorescence (Scale bars = 50 μm).

To evaluate the applicability of using GelTN hydrogel to support cellular infiltration and neovascularisation *in vivo*, GelTN_Lo hydrogels were prepared with or without the pro-angiogenic growth factor fibroblast growth factor 2 (FGF-2) and subcutaneously injected into C57BL/6J mice for a period of 14 days. In these proof of principle experiments, FGF-2 was incorporated into the GelTN hydrogels instead of VEGF-C due to its well-documented pro-angiogenic effects in *in vivo* assays [[95], [96], [97]]. Mice were injected subcutaneously with GelTN_Lo into both sides of the abdomen, as indicated by the arrows in Fig. 10A (left). The injected GelTN hydrogels formed palpable and visible bulges under the skin, indicating successful subcutaneous administration. After 2 weeks, the mice were sacrificed, and the abdominal skin was dissected for collection of GelTN plugs. GelTN plugs remained intact and localised at the injection site within the subcutaneous layer (Fig. 10A right). Histological analysis of GelTN hydrogel plugs was performed using haematoxylin and eosin (H&E) staining. Fig. 10B shows representative H&E-stained images of growth factor-free GelTN (NC) and FGF-2 containing GelTN hydrogels. In the NC controls, limited cell infiltration was observed (Fig. 10B and C). In contrast, FGF-2 containing GelTN hydrogels exhibited substantial cell infiltration, with a significant increase in cell density adjacent to, and within, the hydrogel matrix, compared to NC hydrogels (Fig. 10BandC). Red blood cell-containing perfused neo-vessels, as shown in the magnified inset, were observed in FGF-2 containing GelTN hydrogels (Fig. 10D). Additional representative images of vascular structures are shown in Fig. S8, where red blood cell-filled vessel-like structures were consistently observed across all plugs. To confirm the presence of endothelial cell-lined neo-vessels within the GelTN plugs, sections were immuno-stained using antibodies to the endothelial membrane marker CD31 (Fig. 10E). In addition, FLT4 (VEGFR3) staining revealed LEC signal within the hydrogel region, further supporting the formation of vascularised and lymphatic structures (Fig. 10F).

Compared to a previous study by Koshy et al. where significant cell infiltration was observed after 8 weeks [29], the optimised properties of GelTN in this study facilitated cell and vessel infiltration within just 2 weeks. Our GelTN hydrogel model, incorporating FGF-2, significantly increased cell infiltration and showed the presence of endothelial lined neo-vessels containing red blood cells. On average, 3–4 vascular structures were observed per GelTN plug (n = 4 plugs), indicating that vascularisation was reproducibly achieved in this model even at this early timepoint. Nevertheless, future modifications can be explored to enhance its performance for more effective vascular tissue engineering applications. These include (1) fine-tuning the mechanical properties of GelTN hydrogel (e.g. ligand density), (2) compositional modifications, such as incorporating additional ECM proteins (e.g. fibronectin, laminin) to better mimic the native tissue environment, and (3) combined encapsulation of multiple growth factors (such as VEGF-C, VEGF-A and FGF-2), which might synergistically promote more extensive vascularisation and lymphangiogenesis within the GelTN matrix, as suggested by Kim et al. [107].

It is also important to note that the vascularisation observed in our GelTN was not as extensive as previously observed in studies using Matrigel matrix, a commonly used ECM [51,[98], [99], [100]]. These differences can likely be attributed to the different protein composition and physico-mechanical properties between the two hydrogels. Matrigel is rich in multiple ECM proteins, including laminin, collagen and fibronectin, and naturally supports robust neo-vessel formation and infiltration [101,102]. Additionally, Matrigel's elastic modulus is approximately 450 Pa [103], which is significantly softer compared to GelTN_Lo (∼1.4 kPa), potentially facilitating easier cellular invasion and matrix remodelling. However, despite its widespread use in preclinical studies, Matrigel has significant translational limitations that restrict its application in clinical settings [104,105]. These include batch-to-batch variability, undefined composition, and its derivation from murine sarcomas, which raises concerns regarding xenogeneic components, immunogenicity as well as regulatory approval [105,106]. In contrast, our GelTN bioorthogonal hydrogel platform, with chemically defined and tunable properties, allows more precise control over mechanical stiffness, degradability, and growth factor release kinetics. This makes GelTN a more promising candidate for therapeutic applications where reproducibility, safety, and customisation are critical.

Overall, the subcutaneous model used here serves as a proof-of-concept to demonstrate the angiogenic potential of GelTN *in vivo*. The presence of FLT4^+^ and CD31^+^ vascular structures and perfused neo-vessels within the hydrogel highlights the capacity of GelTN to support vascularisation *in vivo*. We recognise that this approach does not fully replicate disease-relevant contexts where lymphangiogenesis plays a pivotal role. Future studies incorporating extended time points and lymphatic-specific markers (e.g., LYVE-1, PROX1) will be important to further characterise the function of infiltrating vessels, in disease-context models, such as lymphoedema or wound healing.

## 4 Conclusions

The establishment of a 3D *in vitro* lymphangiogenesis model using bioorthogonal click-crosslinked gelatin hydrogels provides a robust platform for replicating critical aspects of cell-ECM interactions and HDLEC sprouting. Our model also enables the investigation of underlying mechanotransduction pathways critical to lymphangiogenesis. Our findings demonstrate that softer GelTN hydrogels (6 % w/v) significantly enhance HDLEC responsiveness to VEGF-C and promote robust and reproducible sprout formation. Importantly, we identified critical roles for MMPs, particularly MMP14, and integrin receptors, β3 – highlighted for the first time in this context – and α5β1, in mediating HDLEC sprouting through dynamic cell-ECM interactions within GelTN hydrogels. Moreover, the controlled encapsulation and sustained release of bioactive VEGF-C from injectable GelTN hydrogels underline their translational potential for therapeutic lymphangiogenesis applications. Preliminary *in vivo* studies demonstrated the capability of GelTN hydrogels to support growth factor-dependent cellular infiltration and neovascularisation. Thus, GelTN hydrogels offer a robust tool for studying lymphangiogenesis *in vitro* and *in vivo*, with the flexibility to adapt to various research needs. The continued development and optimisation of this model will contribute to advancing tissue engineering applications, paving the way for future exploration into targeted regenerative medicine strategies aimed at addressing various lymphatic vessel anomalies and vascular-related pathologies.

## CRediT authorship contribution statement

**Dana E. Al-Ansari:** Writing – original draft, Visualization, Validation, Resources, Methodology, Investigation, Funding acquisition, Formal analysis. **Yangshuo Hu:** Writing – review & editing, Validation, Methodology, Investigation, Formal analysis. **Nicola Contessi Negrini:** Writing – review & editing, Methodology, Investigation. **Daisy Jones:** Validation, Methodology. **Graeme M. Birdsey:** Writing – original draft, Validation, Supervision, Resources, Project administration, Methodology, Investigation, Funding acquisition, Formal analysis, Conceptualization. **Adam D. Celiz:** Writing – original draft, Validation, Supervision, Resources, Project administration, Methodology, Investigation, Funding acquisition, Formal analysis, Conceptualization.

## Declaration of competing interest

The authors declare that they have no known competing financial interests or personal relationships that could have appeared to influence the work reported in this paper.

## Data Availability

Data will be made available on request.
