# Supplementary material for: Three-dimensional modelling of lymphangiogenesis in-vitro using bioorthogonal click-crosslinked gelatin hydrogels
Source: Mater Today Bio. 2025 Oct 1;35:102367. doi: 10.1016/j.mtbio.2025.102367 (PMC12516055; doi:10.1016/j.mtbio.2025.102367)
Supplement: Multimedia component 1 [file mmc1.docx]

**
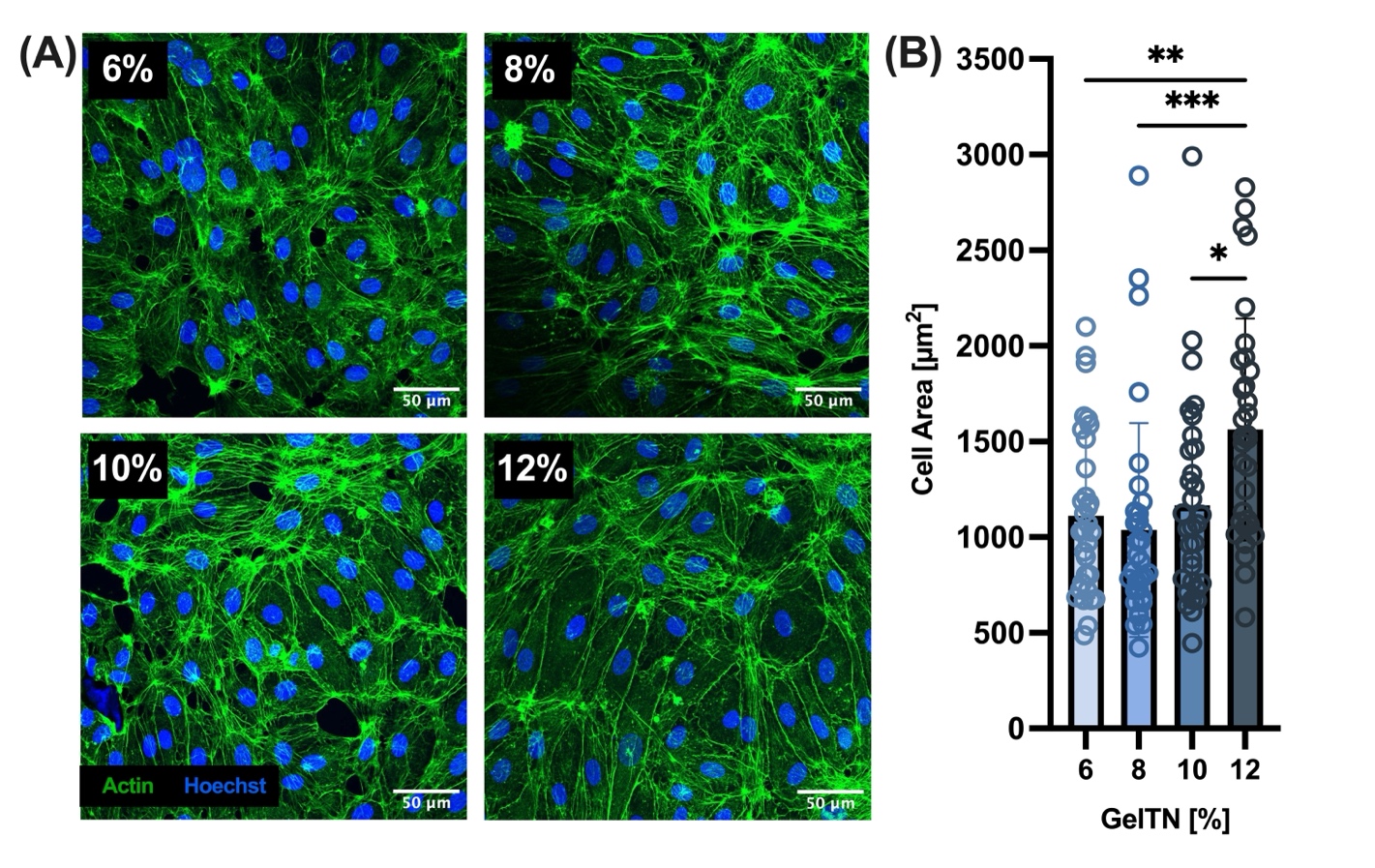
SUPPLEMENTARY**

**Figure S1 Adhesion and spreading of HDLEC at different concentrations of GelTN. (A)** Confocal images showing maximum projection of HDLEC top-seeded on GelTN hydrogels (6 – 12%), stained with DAPI (nucleus; blue) and Phalloidin (actin; green). Images acquired at 40X magnification. Scale bar = 50 µm. **(B)** Quantification of the HDLEC area seeded on GelTN hydrogels. Data are presented as mean ± SD; (*p<0.05, **p<0.01 and ***p<0.001).


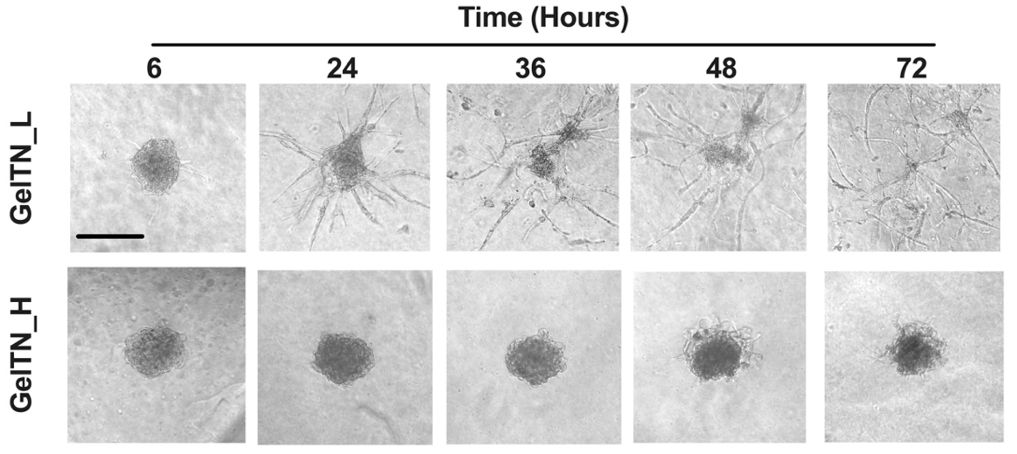


**(A)**

**(B)**

**(C)**

**Figure S2 Sprouting of HDLEC spheroids in GelTN at different concentrations. (A)** Young’s modulus calculated for freshly prepared vs. swollen GelTN hydrogels (6% and 12% w/v; n = 3).  **(B)** Quantification of HDLEC spheroids core area. **(C)** Representative brightfield time-lapse images of HDLEC spheroids cultured in GelTN_Lo (top panel) and GelTN_Hi (bottom panel) for 6, 24, 36, 48, and 72 h in the presence of 50 ng/mL VEGF-C (scale bar = 100 µm). Data are presented as mean ± SD.

**
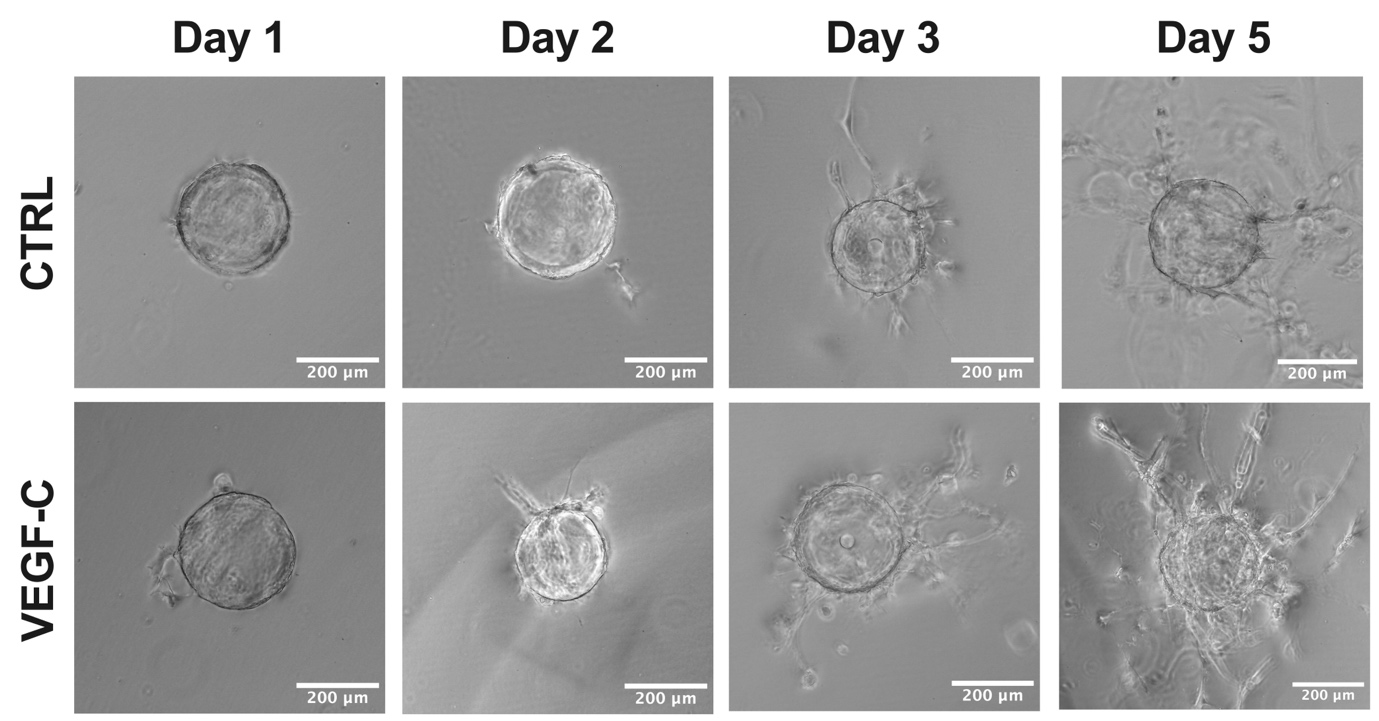
**

**Figure S3 Brightfield microscopic images of HDLEC sprouts, emerging from microcarrier beads embedded in GelTN_Lo, at different time-points.** HDLEC coated beads embedded in GelTN_Lo in the presence and absence of 200 ng/mL VEGF-C. Scale bare = 200 µm, n=4.

**
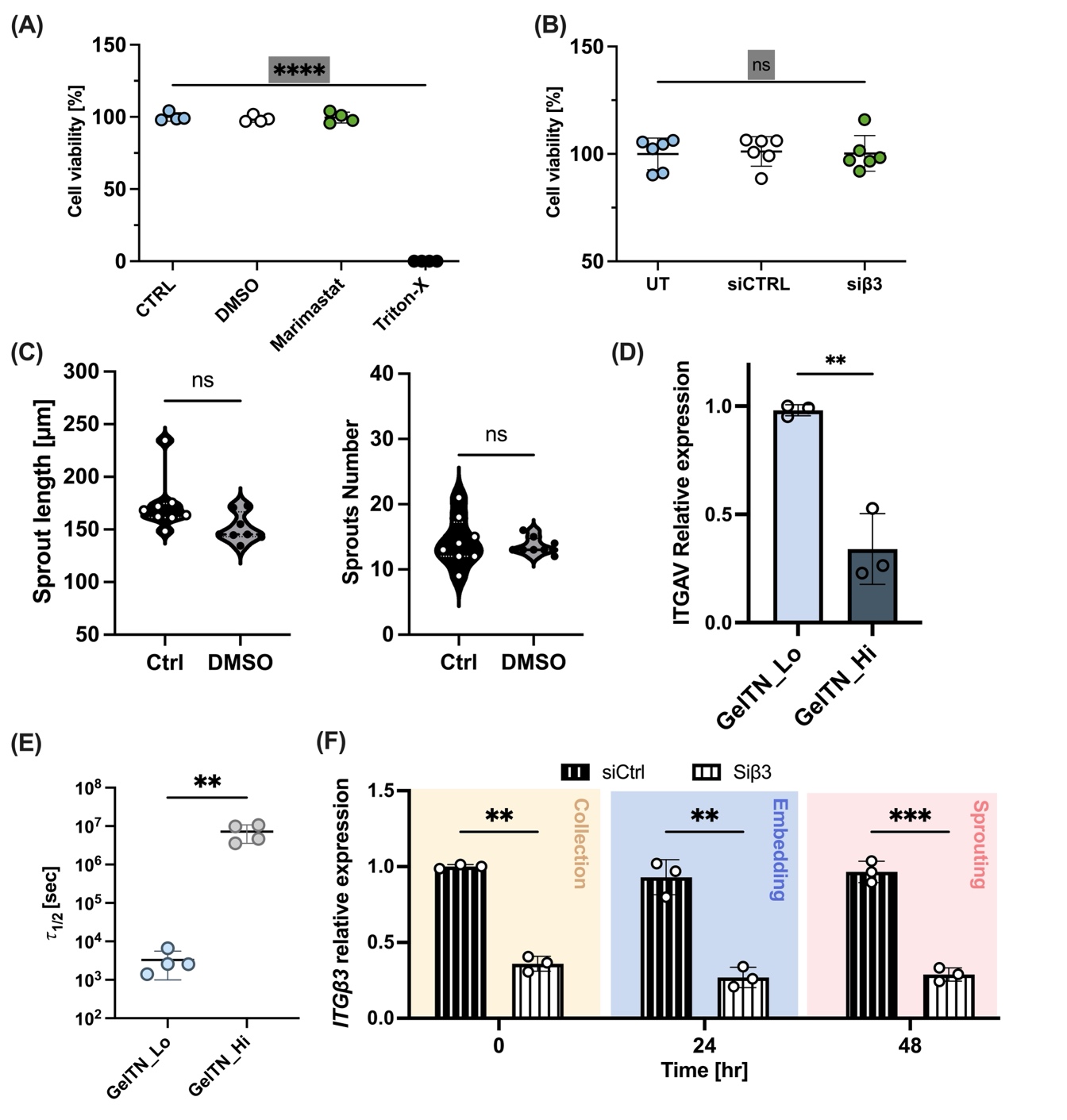

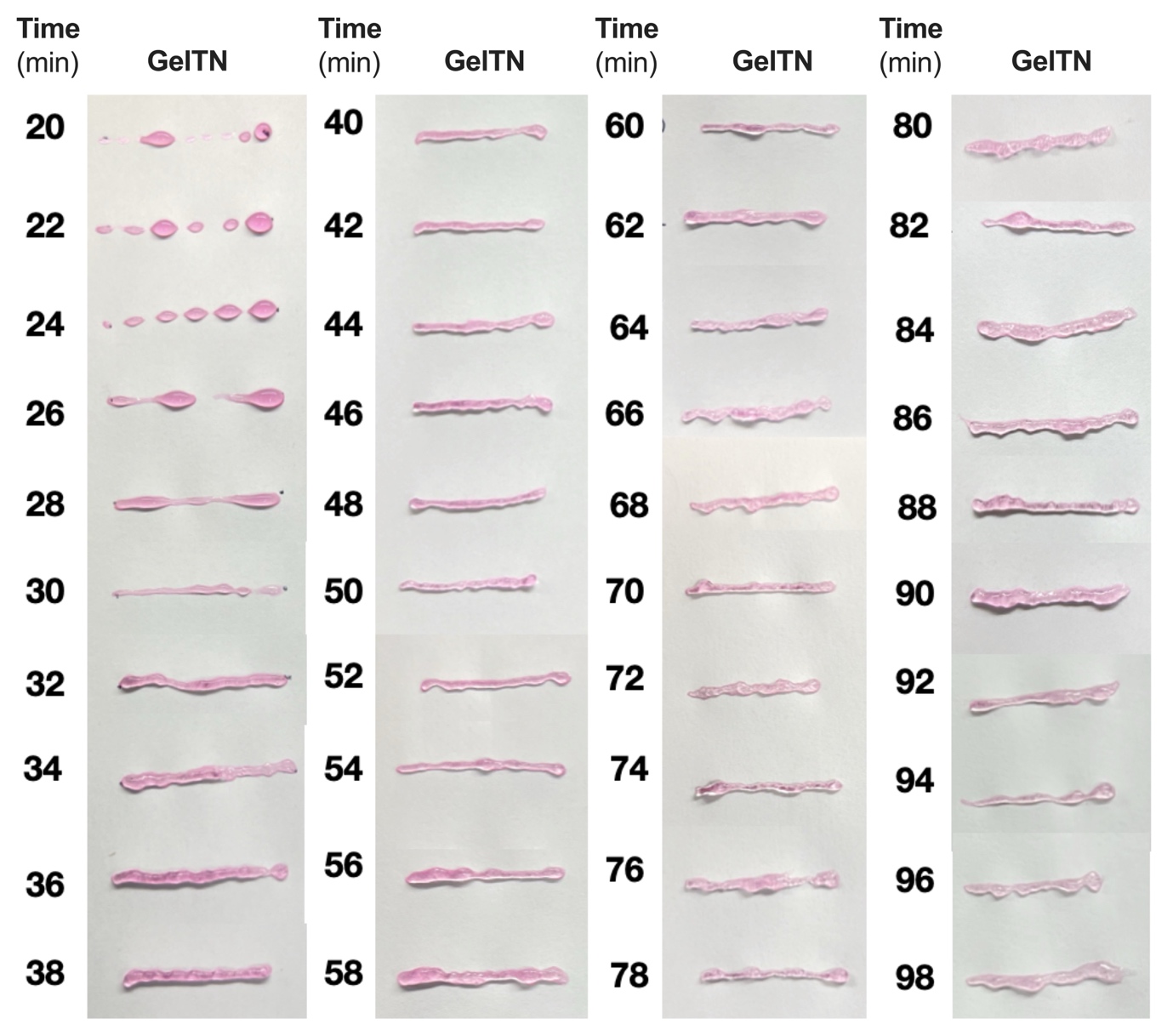
**

**Figure S4 Mechanical regulation of HDLEC lymphangiogenic activity in GelTN_1.4 and GelTN_5.4 kPa hydrogels via matrix metalloproteases (MMPs) and integrins.** Assessment of HDLEC viability post **(A)** Marimastat and **(B)** siβ3 treatments using AlamarBlue assay. Triton-X was used as a positive control for toxicity and DMSO as a vehicle control. **(C)** Quantification of HDLEC spheroid sprout length (left) and number (right) in 6% GelTN in the presence and absence of the vehicle control (DMSO). **(D)** The relative expression of ITGαV integrins in HDLEC 72 hours post hydrogel encapsulation (n = 3). **(E)** Relaxation time of GelTN_Lo or GelTN_Hi (τ1/2). **(F)** HDLEC were treated with siCtrl or siβ3 for 24 hours followed by media change. RT-qPCR of ITGβ3 relative expression at different time-points (0, 24 and 48 hours) post siRNA treatment (n = 3). Each time-point corresponds to a different stage of the sprouting assay timeline: 0 hr = HDLEC collection for spheroids generation, 24 hr: HDLEC spheroids embedding in GelTN hydrogels and 48 hr: HDLEC sprouts imaging. Unpaired t-test was used to analyse the differences between gene expression in siCtrl and siβ3 at each time-point. Data are represented as mean ± SD. **p<0.01 and ***p<0.001.

**Figure S5 Qualitative assessment of GelTN ejectability.** Representative image of GelTN (GelTN_Lo; 6%) injected at different timepoints (20 – 98 min) post-crosslinking, on a flat surface using 1 mL syringe fitted with a 26G’ ½’’ needle.

**
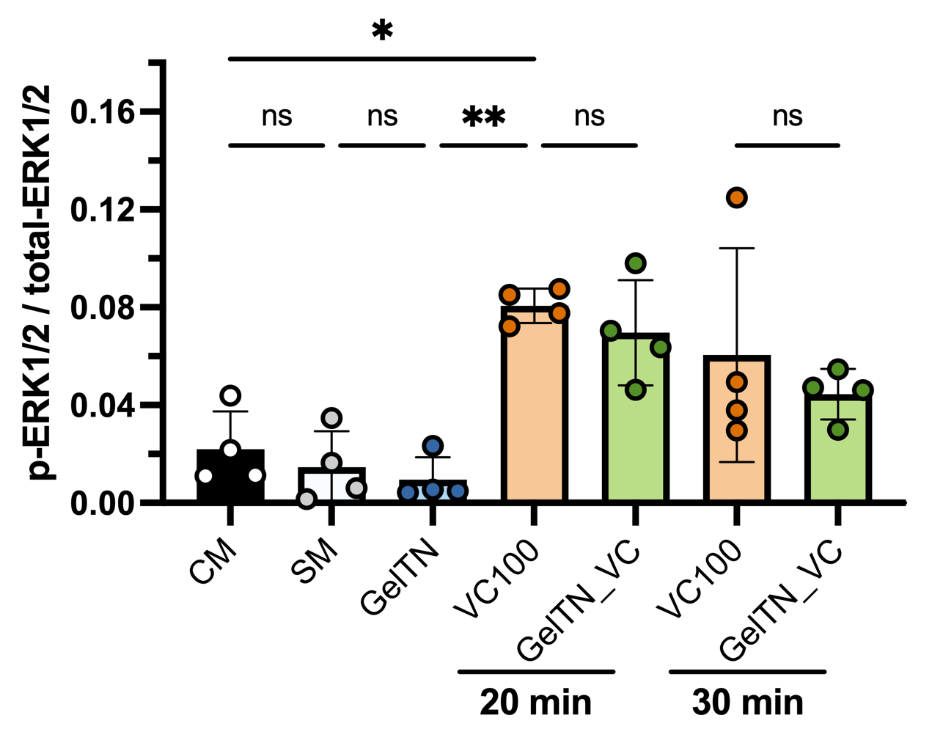
**

**Figure S7 Assessment of long-term bioactivity of VEGF-C released from GelTN hydrogels.** HDLEC were treated with GelTN supernatants collected at different time points (Day 1–10) and assessed for metabolic activity after 72 hours using a AlamarBlue assay. Ordinary One-Way ANOVA, followed by Dunnett’s comparison was used for analysis. Data are represented as mean ± SD. *p < 0.05, **p < 0.01, ***p < 0.001, and ****p < 0.0001 (n = 3).­

**Figure S6 Quantification of ERK1/2 phosphorylation in HDLEC treated with GelTN_V for 20- and 30-min.** Quantification of p-ERK1/2/total-ERK1/2 ratios in HDLEC treated with supernatants from GelTN_VC, GelTN, VC100, CM and SM, for 20 and 30 min (n = 4). Ordinary One-Way ANOVA, followed by Dunnett’s comparison was used for analysis. Data are represented as mean ± SD. *p < 0.05, **p < 0.01, ***p < 0.001, and ****p < 0.0001.­

**
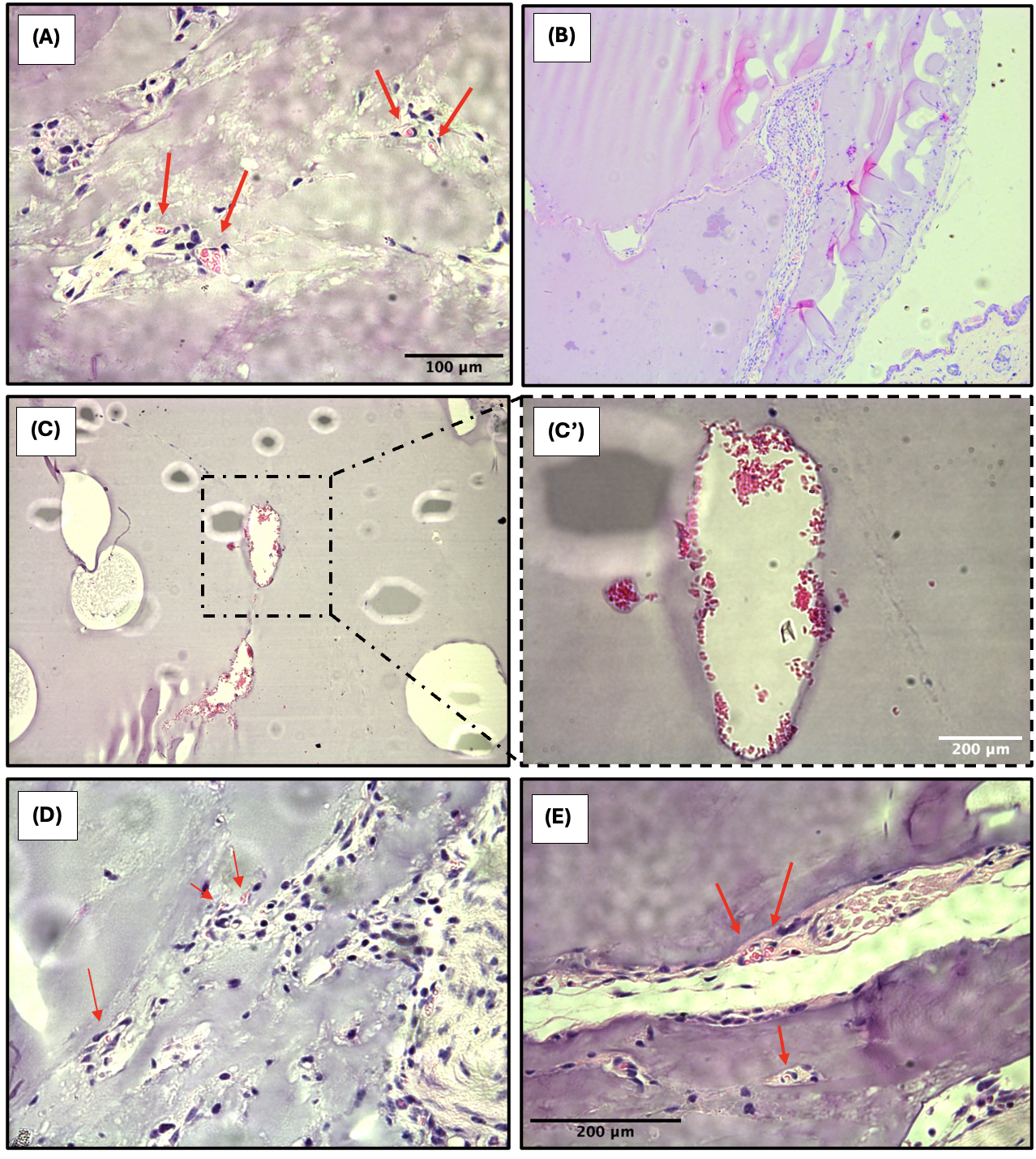
**

**Figure S8 Vascular infiltration within FGF-loaded GelTN hydrogels.**Representative H&E-stained sections from FGF-2-containing GelTN hydrogels plugs 2 weeks post-subcutaneous implantation in mice (n = 4). Red arrows indicate cellular structures resembling blood vessel cross-sections with red blood cells and surrounding endothelial lining. In panel C, the dashed box highlights a perfused vessel within the hydrogel, which is shown at higher magnification in panel C’.

**Table S1. List of primers used for RT-qPCR analysis.**

| **Gene** | **Forward 5’ – 3’** | **Reverse 5’ – 3’** |
| --- | --- | --- |
| GAPDH | CAAGGTCATCCATGACAACTTTG | GGGCCATCCACAGTCTTCTG |
| PROX1 | TACGCACGTCAAGCCATCAA | CAGGAATCTCTCTGGAACCTCA |
| VEGFR3 | GACTGTGGCTCTGCCTGG | GTCCTCGCTGTCCTTGTCTC |
| MMP14 | GCAGAAGTTTTACGGCTTGCAA | CCTTCGAACATTGGCCTTGAT |
| MMP9 | ATCCAGTTTGGTGTCGCGGAGC | GAAGGGGAAGACGCACAGCT |
| MMP2 | CTCAGATCCGTGGTGAGATCT | CTTTGGTTCTCCAGCTTCAGG |
| Integrin-β1 | ACTGATTGGCTGGAGGAATG | AACAATGCCACCAAGTTTCC |
| Integrin-β3 | TTCAATGCCACCTGCCTCAA | TTGGCCTCAATGCTGAAGCTC |
| Integrin-αV | CCCCGAGGGAAGTTACTTCG | ATGGATCATCCTTGGCATAATCTC |
| Integrin-α5 | CACTGGCCATGATGAGTTTG | CGATGGCCACATCATTGTAG |

| **Gene** | **Forward 5’ – 3’** | **Reverse 5’ – 3’** |
| --- | --- | --- |
| GAPDH | CAAGGTCATCCATGACAACTTTG | GGGCCATCCACAGTCTTCTG |
| PROX1 | TACGCACGTCAAGCCATCAA | CAGGAATCTCTCTGGAACCTCA |
| VEGFR3 | GACTGTGGCTCTGCCTGG | GTCCTCGCTGTCCTTGTCTC |
| MMP14 | GCAGAAGTTTTACGGCTTGCAA | CCTTCGAACATTGGCCTTGAT |
| MMP9 | ATCCAGTTTGGTGTCGCGGAGC | GAAGGGGAAGACGCACAGCT |
| MMP2 | CTCAGATCCGTGGTGAGATCT | CTTTGGTTCTCCAGCTTCAGG |
| Integrin-β1 | ACTGATTGGCTGGAGGAATG | AACAATGCCACCAAGTTTCC |
| Integrin-β3 | TTCAATGCCACCTGCCTCAA | TTGGCCTCAATGCTGAAGCTC |
| Integrin-αV | CCCCGAGGGAAGTTACTTCG | ATGGATCATCCTTGGCATAATCTC |
| Integrin-α5 | CACTGGCCATGATGAGTTTG | CGATGGCCACATCATTGTAG |
